# Lignin Mimicking Protein Methylation Augments the Efficacy of FOLFOX Chemotherapy by Anchoring Thymidylate Synthase

**DOI:** 10.1002/adma.202507040

**Published:** 2026-08-17

**Authors:** Shiyao Song, Yan‐Lai Tang, Xiaoting Liu, Jiayi Zheng, Chong Wu, Tang Li, Junhong Ye, Dianmin Xia, Yong Mei, Jingxuan Ma, Libin Huang, Xuequn Luo, Liheng Chen, Yanlin Qin, Weiling He

**Affiliations:** ^1^ Department of Pediatrics The First Affiliated Hospital Sun Yat‐sen University Guangzhou Guangdong China; ^2^ Department of Gastrointestinal Surgery The First Affiliated Hospital Sun Yat‐sen University Guangzhou Guangdong China; ^3^ Department of Gastrointestinal Surgery School of Medicine Xiang'an Hospital of Xiamen University Xiamen University Xiamen Fujian China; ^4^ Department of Immunology Zhongshan School of Medicine Sun Yat‐sen University Guangzhou Guangdong China; ^5^ Guangdong Provincial Key Laboratory of Plant Resources Biorefinery School of Chemical Engineering and Light Industry Guangdong University of Technology Guangzhou China; ^6^ Guangdong Medical University Affiliated Hospital Guangdong Medical University Zhanjiang Guangdong China; ^7^ Department of Oncology The First Affiliated Hospital Jinan University Guangzhou Guangdong China; ^8^ Department of Hepatobiliary Surgery The Affiliated Hospital of Guizhou Medical University Guiyang China; ^9^ West China School of Medicine Sichuan University Chengdu Sichuan China

**Keywords:** charge reversal, colorectal cancer, FOLFOX, lignin, nanoparticles

## Abstract

FOLFOX has served as the standard chemotherapy regimen for advanced stages, specifically in the treatment of pancreatic, colorectal, and bladder cancers. However, the issues of excessive toxicity and prolonged treatment cycles persist. To address this limitation, we developed a novel methylated amino acid‐modified lignin inspired by biological methylation, creating a pH‐responsive charge‐reversal drug delivery system for FOLFOX chemotherapy. This system stabilizes thymidylate synthase (TS) and combines with deoxyuridine monophosphate (dUMP) to demonstrate significantly improved therapeutic outcomes of FOLFOX against colorectal cancer (CRC). The FOLFOX‐loaded nanodrug showed targeted antitumor activity against CRC and induced tumor cell death through multiple mechanisms, including apoptosis, necrosis, cell cycle regulation, DNA damage, and reactive oxygen species generation. Furthermore, the nano‐formulation activated immune responses to enhance chemotherapeutic potency and effectively reducing renal toxicity associated with chemotherapy drugs. These findings suggest that protein methylation‐mimicked lignin could be a valuable addition to current first‐line chemotherapy options and holds promise for enhancing chemotherapy against solid tumors.

## Graphical abstract

Lignin‐mimicking protein methylation can enhance the efficacy and mitigate the toxicity of the classic FOLFOX chemotherapy regimen.

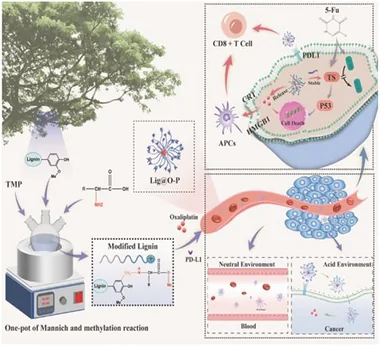

## 1 Main

The FOLFOX regimen, a standard adjuvant chemotherapy (ACT) for pancreatic, colorectal, and bladder cancers [1], including three drugs: folinic acid (FnA), 5‐fluorouracil (5‐Fu), and oxaliplatin (OXA) [2]. In clinical practice, patients receive intravenous (i.v.) infusions of oxaliplatin and FnA simultaneously, followed by the i.v. administration of 5‐Fu over a 2‐week period [3]. Despite improvements in FOLFOX application methods and enhanced survival rates, key challenges remain, including low efficacy, dose‐limiting side effects, and prolonged treatment courses [4]. These issues contribute to a high incidence of recurrence and metastasis, poor quality of life, and increased costs [5]. Therefore, a more specific delivery scheme for FOLFOX is needed.

Biological methylation is a crucial chemical modification process within living organisms, wherein a methyl group (─CH_3_) is added to DNA, RNA, or proteins [6]. This complex and essential process influences health and development by regulating gene expression and maintaining genome stability. The methylation of protein amino acid (AA) residues, in particular, plays a significant role and has attracted extensive interest from researchers [7]. Abnormal methylation patterns are often associated with various diseases, especially cancer [6]. For instance, excessive methylation can silence tumor suppressor genes in cancer cells, contributing to tumor development [8]. Small molecules or drugs can target and interfere with abnormal methylation in tumor cells to restore normal gene expression [9]. For example, FnA works synergistically with 5‐Fu as a thymidylate synthase (TS) inhibitor, inhibiting the methylation of deoxyuridine monophosphate (dUMP) to form thymidine monophosphate (dTMP), thereby suppressing cancers [10]. These features suggest that protein methylation patterns can be utilized to create delivery vectors, thereby enhancing the biological activity of nanomedicine.

In plants, methylation also plays a crucial role in the biosynthesis of lignin [11]. The methylation of lignin alters its electronic density distribution and hydrogen bond network, affecting its hydrophilicity and hydrophobicity, consequently impacting water transport [12]. Due to its controllable hydrophilicity and hydrophobicity, lignin can achieve stable dispersion in water through self‐assembly, forming lignin nanoparticles [13]. Notably, as the most abundant biodegradable phenolic polymer in nature, lignin nanoparticles offer numerous advantages, including excellent biocompatibility, high‐efficiency drug loading, and ease of functional modification [14, 15, 16]. However, while natural lignin exhibits intrinsic bioactivities such as antioxidant effects and potential chondroprotective roles [17, 18], its unmodified form lacks targeted therapeutic efficacy for complex diseases.

To fully utilize the inherent advantages of lignin while endowing it with the ability to treat human diseases, this study modifies lignin by mimicking protein methylation and applies it to cancer treatment. First, lignin is AA‐modified to provide AA residues similar to those of proteins, enhancing its biocompatibility in vivo. It then undergoes methylation to regulate its electronic density distribution, achieving charge reversal. The resulting lignin nanoparticles can easily load oxaliplatin and PD‐L1 to form a novel lignin‐based nano‐agent (Lig@O‐P). We verified the targeted delivery capability of this modified lignin both in vitro and in vivo. We found that Lig@O‐P induces apoptosis, causes DNA damage, arrests the cell cycle, and produces ROS and exerts potent antitumor effects against colorectal cancer (CRC). Notably, Lig@O‐P can bind to TS and dUMP, enhancing FOLFOX chemoprevention. In vivo results further confirmed that Lig@O‐P effectively inhibits tumor growth while demonstrating considerable long‐term biocompatibility in healthy tissues. Additionally, it significantly reduces the kidney toxicity associated with chemotherapy drugs and demonstrates powerful immune activation, further enhancing the FOLFOX therapeutic effect. In summary, we propose a novel lignin‐based FOLFOX nanomedicine that offers a new first‐line chemotherapy drug delivery scheme, reducing patient side effects and enhancing treatment efficacy. Furthermore, it provides new insights into applying complex macromolecular polymers, such as lignin, in treating various solid tumors.

## 2 Results

### 2.1 Fabrication and Characterization of Amino Acids and Methylation‐Modified Lignin Nanoparticles

Our previous research has demonstrated that the methylation of AA‐functionalized lignin can be efficiently achieved through a combination of the Mannich reaction and methylation [12]. Specifically, as illustrated in Figure 1a, lignin is first reacted with AAs to produce AA‐functionalized lignin, followed by the addition of trimethyl phosphate (TMP) in the same reactor for in situ methylation. To determine whether this process regulates electron distribution in a manner similar to protein methylation, we employed density functional theory (DFT) calculations to analyze the electrostatic potential (ESP) distribution. As shown in Figures 1b and S1, the unmodified lignin (guaiacylglycerol‐beta‐guaiacyl ether, GGE) exhibits a narrow ESP distribution, lacking regions with significantly positive or negative ESP. However, after AA functionalization, the ESP distribution of lignin (ARG‐GGE) broadens, with increased absolute values of both positive and negative potentials. Following further methylation, the ESP distribution of lignin (ARG‐GGE‐Me) shows minimal change, but the positive potential tends to increase. These findings indicate that the methylation of AA‐modified lignin induces a redistribution of electrons, expanding the range of ESP. This suggests that the modification enhances the tunable properties of lignin, facilitating the loading of various drugs or active substances, thereby improving its potential for biomedical applications.

**FIGURE 1:**
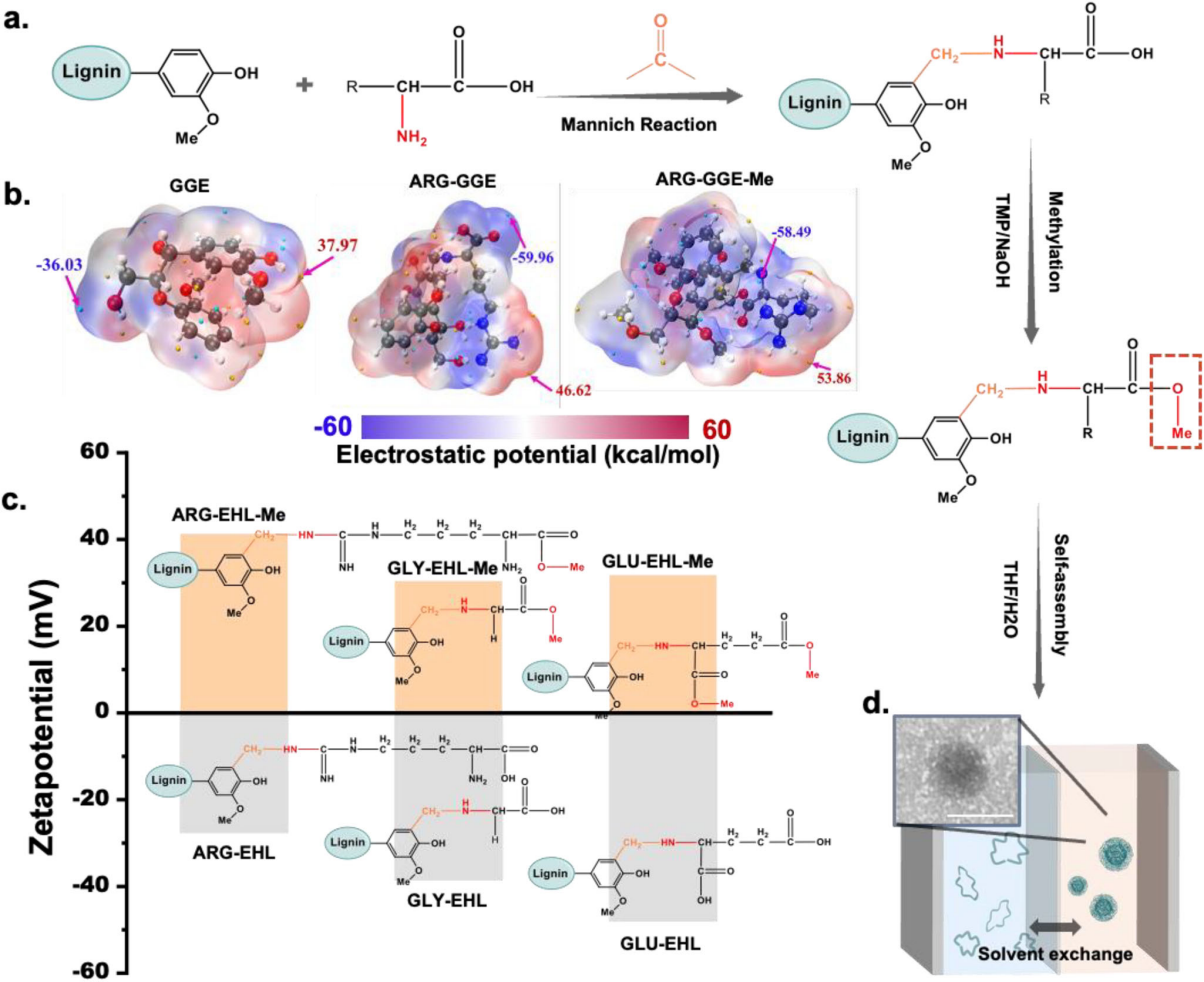
Schematic and characterization of lignin modified by amino acid and methylation. (a) Illustration of the lignin‐modified procedure. (b) The electrostatic potential (ESP) distribution analyzed from density functional theory (DFT) calculations in different modified lignins. (c) The zeta potentials of lignin modified by amino acid and methylation. (d) The morphology of ARG‐EHL‐Me NPs by TEM. Bar indicates 200 nm scale. ARG: arginine; GLU: glutamic; GLY: glycine; GGE: guaiacylglycerol‐beta‐guaiacyl ether, unmodified lignin.

To identify the most suitable AA‐methylated lignin for specific applications, we compared the effects of various AA modifications on enzymatic hydrolysis lignin (EHL), including acidic (GLU, *pI* = 3.2), basic (ARG, *pI* = 10.8), and nonpolar (GLY, *pI* = 6.8) AAs. The successful synthesis of the AA and methylation‐modified lignin was confirmed by FTIR analysis (Figure S2). To further characterize the polymer state after modification, the molecular weight distribution of lignin before and after modification was analyzed by GPC/SEC (Figure S3). Compared with the starting GGE (Mn = 1044, Mw = 3046, *Đ* = 2.92), ARG‐GGE showed increased Mn (1285) and Mw (3596), supporting successful arginine (ARG) grafting onto the lignin backbone. After methylation, ARG‐GGE‐Me exhibited lower Mn (931), Mw (2150), and dispersity (*Đ* = 2.31) than ARG‐GGE, indicating a shift toward lower molecular‐weight fractions together with a narrower molecular‐weight distribution. These results provide additional evidence for the polymer‐state evolution during modification and support the acceptable homogeneity of the modified lignin used for subsequent nanoparticle fabrication. We also assessed the charge properties of the differently modified lignin at neutral pH (Figure 1c). All three AAs exhibited strong electronegativity after modification, with charges of −48, −38.5, and −27.6 mV, respectively, and all measurements were conducted in phosphate‐buffered saline (PBS, pH 7.4) to maintain physiological relevance. Following methylation, ARG demonstrated the highest positive charge (+ 41.3 mV), while glutamic acid (GLU) and glycine (GLY) showed comparable positive charges (+ 31.8 and + 30.4 mV, respectively). This can be attributed to the higher number of polar guanidine groups in ARG, which facilitate the protonation of amine groups and the acquisition of a positive charge. To determine the most appropriate colloidal spheres for biological applications, we examined the hydrodynamic size of the modified lignin at different pH values. The results indicated that ARG‐modified EHL followed by methylation (ARG‐EHL‐Me) exhibited a suitable nano‐size and PDI index in a neutral environment, but unstable in acidic conditions, suggesting disintegration of the nanostructure as pH decreases (Figure S5). Consequently, ARG‐EHL‐Me was selected for drug delivery in subsequent studies due to its highest positive charge, stability in neutral environments such as the bloodstream, and effective release in acidic environments like tumors. However, the hydrated particle size of the synthesized ARG‐methylated lignin was still too large under neutral conditions (378.3 nm). Therefore, we designed experiments to optimize the reaction conditions (including organic solvent, TMP concentration, NaOH, and ARG concentration) to reduce the hydrated particle size. The results showed that using 70% THF as the organic solvent, 8 mL of TMP, 0.5 g of NaOH, and 0.5 g of ARG resulted in the smallest diameter under neutral conditions (Figure S6). The final nanoparticle size was 74.57 nm, and its morphology is shown in Figure 1d.

The 2D‐HSQC NMR spectrum of lignin can be divided into aliphatic, side chain, and aromatic regions. Since the aliphatic regions does not contain important information about the lignin structure, it is not discussed here. In the side chain region (*δ*C/*δ*H 40.0–90.0/2.50–6.00), *β*‐aryl ethers (*β*‐O‐4, A), resinols (*β*–β, B), and phenylcoumarans (*β*‐5, C) substructures were detected. Methoxy (OMe, *δ*C/*δ*H 55.9/3.73) was present in all lignin samples. In the aromatic region (*δ*C/*δ*H 90.0–150.0/6.00–8.00), the peaks at G2, G5, and G6 sites of lignin were weakened, and the peaks of the Cα of AAs and the methoxy group at the end of amino acids (AAOMe) were detected after methylation and AA modification, which proved that AAs were successfully grafted onto the lignin. At the same time, a peak indicating methylation of lignin phenyl phenolic hydroxyl groups (ArOMe)was detected, further confirms the methylation modification of lignin phenolic groups (Figure S4).

### 2.2 Development of Modified Lignin Nanoparticles for Targeted Delivery of FOLFOX

To utilize novel ARG‐EHL‐Me nanoparticles for delivering modified FOLFOX regimen to tumors, and in alignment with the administration protocol of FOLFOX (injecting oxaliplatin first, followed by 5‐fluorouracil), we synthesized PD‐L1‐targeted nanoparticles loaded with oxaliplatin, designated as Lig@O‐P. The specific synthesis method is illustrated in Figure 2a. Initially, ARG‐methylated lignin particles loaded with oxaliplatin were formed using the antisolvent method. Subsequently, the strong positive charge of the modified lignin facilitated the binding of negatively charged PD‐L1 antibodies, resulting in the formation of the final PD‐L1‐targeted nanodrug. To verify the successful loading of oxaliplatin, we analyzed the Fourier‐transform infrared spectroscopy (FT‐IR) spectra of Oxp, Arg‐EHL‐Me, and Arg‐EHL‐Me@Oxp (Figure S7a). The results indicated that the characteristic peaks of oxaliplatin appeared at 2956, 1704, and 1375 cm^−^^1^, corresponding to the N─H and C═O stretching vibrations. Additionally, distinct signals at 1592 and 1120 cm^−^^1^ were associated with the aromatic ester bending vibration and the C─N stretching vibration of Arg‐EHL‐Me. All characteristic bands of Arg‐EHL‐Me@Oxp at 2956, 1704, 1375, 1592, and 1120 cm^−^^1^, representing the stretching vibrations of N─H, C═O, aromatic ester, and C─N, respectively, were present in both the Oxp and Arg‐EHL‐Me spectra, confirming the presence of Oxp and Arg‐EHL‐Me in Arg‐EHL‐Me@Oxp. Pt was subsequently measured by ICP‐MS, further confirming the successful loading of Oxp (Figure S7b).

**FIGURE 2:**
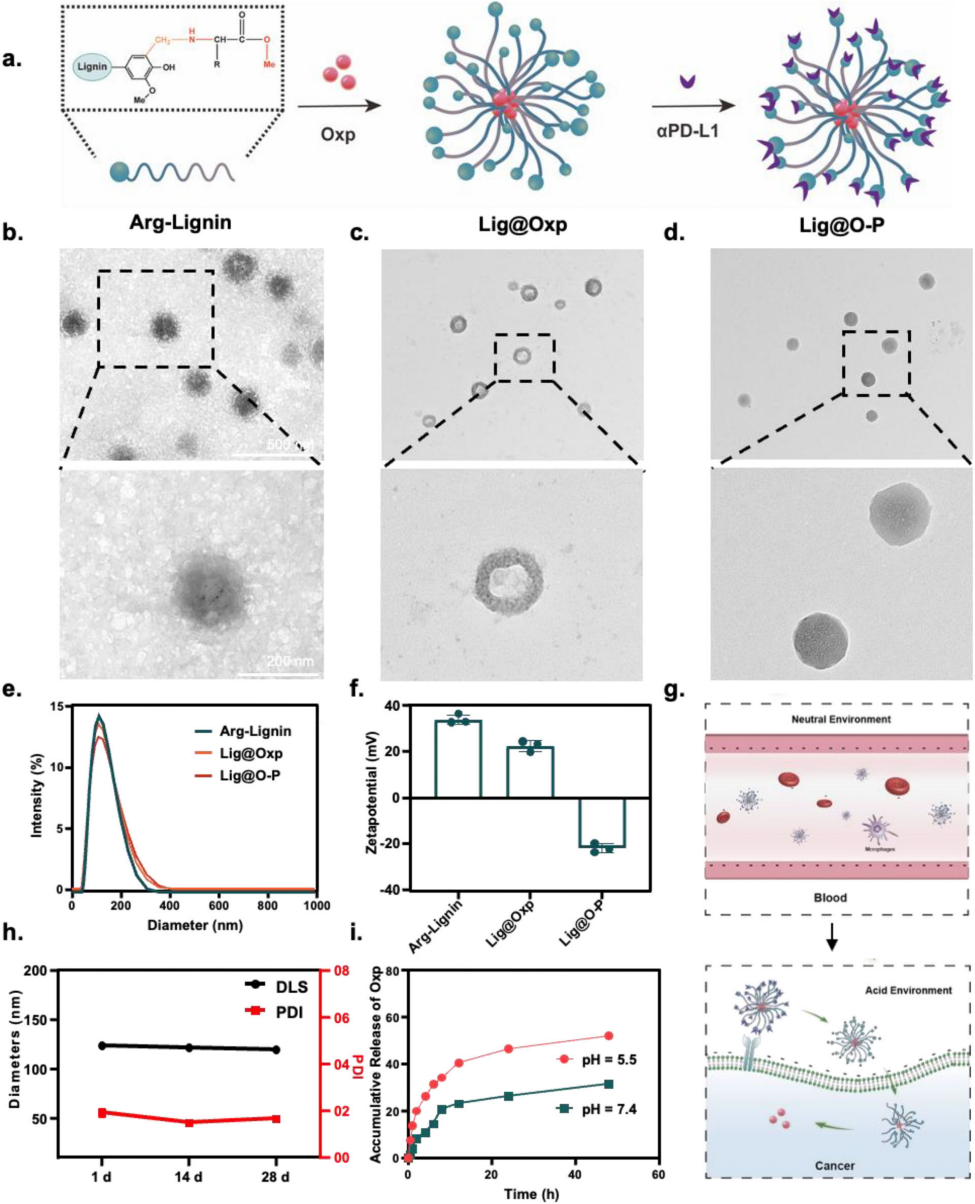
Preparation and characterization of Lignin@Oxp‐αPD‐L1 (Lig@O‐P). (a) Illustration of the Lig@O‐P purification process using Arg‐lignin‐Me. (b–d) The morphology of ARG‐EHL‐Me. (b) Lig@Oxp; (c) Lig@O‐P (d) NPs by transmission electron microscopy. Bar indicates 500 and 200 nm scale, respectively. (e) Size measurements of Arg‐lignin, Lig@Oxp, and Lig@O‐P. (f) Zeta potential measurements of Arg‐lignin, Lig@Oxp, and Lig@O‐P. (g) Proposed schematic illustration of the charge‐reversal behavior of Lig@O‐P during blood circulation and in the acidic tumor‐associated/intracellular environment. The diagram is intended to present a conceptual model of surface shielding, acid‐promoted antibody detachment, and subsequent exposure of the cationic lignin core, rather than a direct real‐time structural observation. (h) Stability of Lig@O‐P diameters and PDI in PBS. (i) The release curves of Lig@O‐P in pH = 5.5 and pH = 7.4. Oxp: οxaliplatin; αPD‐L1: programmed death‐ligand 1 antibody. Data are presented as mean ± SD, *n* = 3 per group.

The morphology of each reaction product was then observed via TEM (Figure 2b–d) and SEM (Figure S8). The results revealed that after loading oxaliplatin, the particles exhibited a distinct putamen structure (Figure 2c), indicating efficient encapsulation of oxaliplatin within the lignin nanoparticles, with an encapsulation efficiency of 44% (Figure S9). After the attachment of the targeted antibody, the putamen structure was replaced by a thick, high‐contrast structure, suggesting a significant payload of PD‐L1 due to the strong positive charge of the material (Figure 2d). This finding was consistent with our zeta potential results, which showed that strongly positive products became negative after loading PD‐L1 (Figure 2f). Additionally, the three products displayed similar diameter sizes, consistent with DLS measurements (Figure 2e). The average hydrated particle sizes were 106, 120, and 121.2 nm, with PDI of 0.13, 0.157, and 0.155, respectively. Consistent with our hypothesis, the zeta potential data demonstrated the charge reversal capability of the material (Figure 2f). A significant number of antibodies were adsorbed onto the material's surface through electrostatic interaction, resulting in a strong negative charge. This modification facilitates effective transport in the bloodstream, reduces rejection by the mononuclear phagocyte system, and minimizes interception by blood vessel walls, thereby extending blood circulation time. Upon reaching the tumor, the pH change triggers the shedding of the surface antibody, exposing the material's strong positive charge. This enhances the penetration depth of nanoparticles within the tumor and promotes the local release of oxaliplatin (Figure 2g). The stability of the material in PBS was also assessed. Remarkably, Lig@O‐P maintained stable particle size and PDI index for 28 days (Figure 2h), In addition, Lig@O‐P also maintained stable hydrodynamic size and PDI in serum‐containing medium, further supporting its colloidal stability under physiologically relevant protein‐rich conditions (Figure S10), likely due to the inherent stability of the lignin structure, which allows the material to remain stable in blood circulation for extended periods. Additionally, the Oxp‐release behavior of Lig@O‐P was compared at pH 7.4 and 5.5, simulating the blood circulation environment and the acidic lysosomal environment in tumors, respectively. As shown in Figure 2i, approximately 20% and 40% of oxaliplatin were released over 12 h under pH 7.4 and pH 5.3 conditions, respectively. This release profile is advantageous for Lig@O‐P nanoparticles, as large amounts of oxaliplatin are rapidly released in the acidic endo‐lysosomal environment following cancer cell uptake. To further support the proposed pH‐responsive surface transformation, fluorescence‐labeled anti‐PD‐L1 was used to evaluate antibody retention on Lig@O‐P after incubation under neutral and acidic conditions. The fluorescence associated with the nanoparticle fraction was lower after incubation at pH 5.5 than at pH 7.4, supporting reduced surface‐retained antibody and acid‐promoted surface de‐shielding under acidic conditions (Figure S11). These results collectively demonstrate the successful synthesis of modified lignin‐based nanomedicine for targeted FOLFOX delivery.

### 2.3 Targeting Ability of Lig@O‐P

The intracellular delivery of Lig@O‐P in vitro was evaluated using fluorescence microscopy, flow cytometry (FCM), ICP‐MS, and bio‐TEM. Rhodamine‐B, known for its strong red fluorescence, was incorporated into the lignin to visualize its intracellular presence. To investigate the intracellular distribution of the nanoparticles, CT26 cells were stained with lysotracker‐FITC after treatment with Rhodamine‐B‐labeled Lig@O‐P. As shown in Figures 3a and S9, a distinct yellow color was observed in Lig@O‐P‐treated cells, compared to those treated with untargeted lignin, indicating that the majority of the nanoparticles were internalized via lysosomal pathways. FCM analyses further demonstrated that Lig@O‐P exhibited stronger binding to cancer cells than both the control (free Rhodamine‐B) and lignin alone (Figures 3c and S12). Additionally, the intracellular uptake of the nanoparticles was time‐dependent, with red fluorescence intensity increasing over time (Figure S13). Co‐localization studies revealed that the nanoparticles primarily accumulated in acidic lysosomes, which is advantageous for enhancing the release of oxaliplatin from the nanoparticles (Figure 3b). This intracellular delivery was also corroborated by the increased Pt concentration detected by ICP‐MS (Figure S14) and the visual evidence provided by bio‐TEM (Figure 3h). Cells treated with Lig@O‐P displayed higher levels of Pt internalization, as shown in Figure S14, and bio‐TEM images clearly depicted the presence of nanoparticles within the cells (Figure 3h). These findings confirm the successful intracellular uptake of Lig@O‐P and its transport through lysosome‐mediated endocytosis.

**FIGURE 3:**
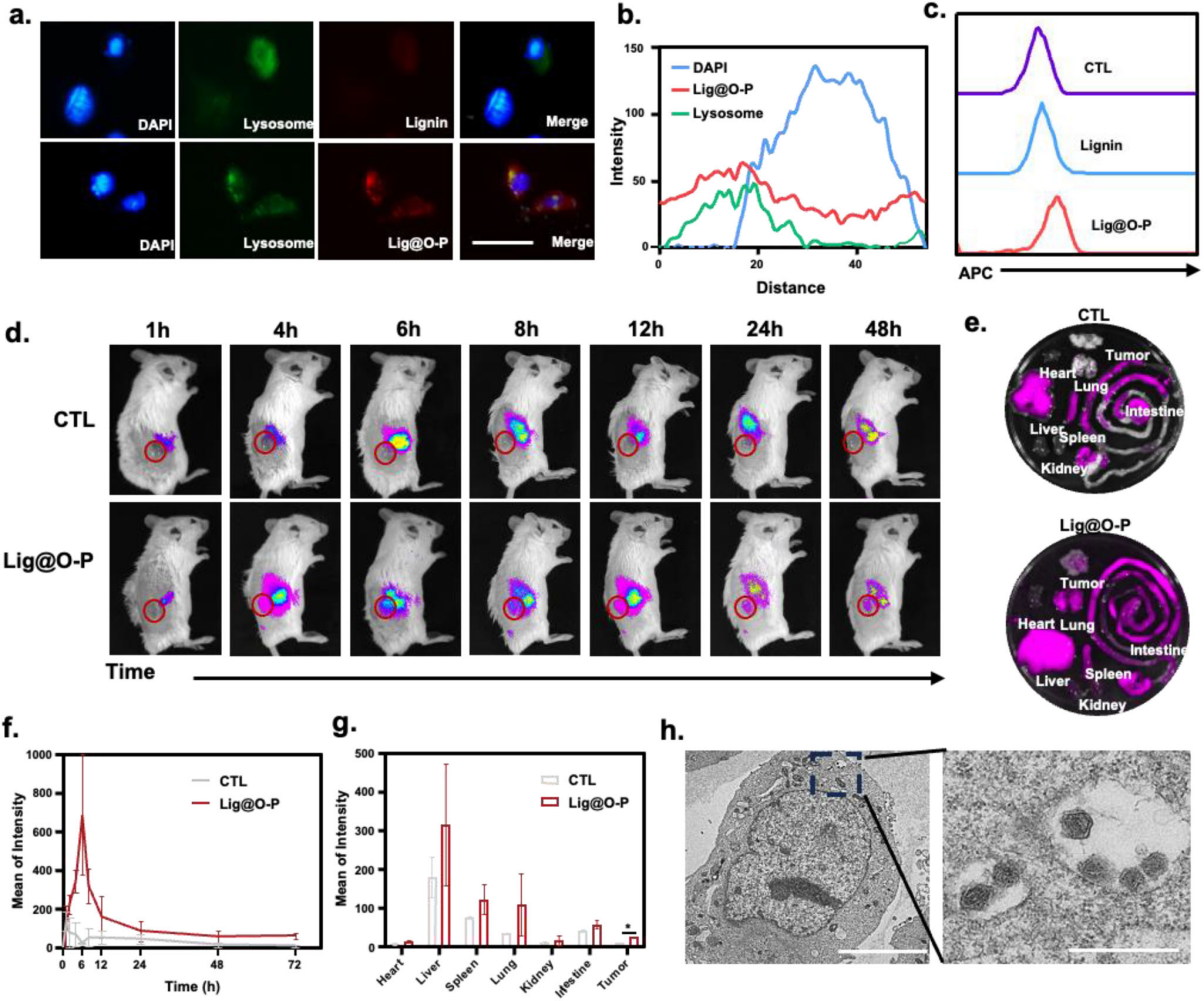
The targeting ability of Lig@O‐P in vitro and in vivo. (a) Confocal microscopy imaging and (b) co‐localization analysis of cancer cell (CT26) uptake for lignin and Lig@O‐P. Intracellular fluorescence is visualized at 6 h upon treatment. Red: Lig@O‐P or lignin (Rhodamine‐B), green: FITC‐lysotrack, lysosome, blue: DAPI, nucleus. Scale bar: 50 µm. (c) FCM analysis of intercellular uptake of Lig@O‐P in CT26 cells. (d, e) In vivo fluorescence biodistribution of ICG‐labeled Lig@O‐P and ICG‐labeled lignin postinjection in CT26 tumor‐bearing mice in different times. (f, g) Ex vivo fluorescence distribution in tumor and major organs at 72 h postinjection. (h) Representative TEM images of intracellular nanoparticles in CT26 cells. CTL: control group; Lig@O‐P: Lig@Oxp‐αPD‐L1. Bar indicates 5 µm and 500 nm scale, respectively.

To further investigate the targeting and distribution of Lig@O‐P in vivo, we assessed the tumor accumulation behavior of ICG‐labeled Lig@O‐P by tracking ICG fluorescence using an IVIS Spectrum imaging system. In CT26‐bearing mice, Lig@O‐P demonstrated a significantly higher tumor uptake rate compared to free ICG at the designated time points (Figure 3d,e). Moreover, ex vivo fluorescence imaging of tumors and other organs 72 h post‐intravenous administration revealed that the fluorescent intensity in the Lig@O‐P group was ten times higher in the tumor compared to the free ICG group (Figure 3f,g). Collectively, these data indicate that Lig@O‐P preferentially targets CRC cells both in vitro and in vivo.

### 2.4 Lig@O‐P Induces Apoptosis, DNA Damage, and ROS Production to Eradicate Colorectal Cancer Cells

The cytotoxic efficacy of Lig@O‐P was assessed using the CCK8 assay. As shown in Figure 4a,b, the IC50 of Lig@O‐P for CT26 (mouse CRC cells) and NCM460 (human normal colonic epithelial cells) was 31.78 nM and 78.92 µg/ml, respectively (Figure 4a). This indicates that Lig@O‐P selectively targets colorectal tumor cells while sparing normal cells. Moreover, Lig@O‐P exhibited stronger cytotoxicity against CRC cells compared to the control, free oxaliplatin, and empty lignin (Figure 4b). Consistent with previous findings, Lig@O‐P‐treated cells showed an increase in LDH release, supporting a lytic form of cell death (Figure 4c). Cellular proliferation analysis by CCK8 assay further confirmed that Lig@O‐P more effectively inhibited cancer cell growth compared to free oxaliplatin (Figure 4d). The cytotoxic effect of Lig@O‐P was also evaluated through live/dead cell staining with calcein‐AM and propidium iodide (PI), as well as FCM with annexin V‐FITC and PI staining. Distinct cell death was observed in live/dead staining images after Lig@O‐P treatment, as compared to PBS and oxaliplatin‐treated cells (Figures 4e and S15a). FCM data revealed that the mortality rate of CT26 cells was 3.2%, 1.47%, 5.17%, and 17.66% in the control, free oxaliplatin, Lig@Oxp, and Lig@O‐P groups, respectively (Figures 4f and S15b). Additionally, Western blot analysis showed that after Lig@O‐P treatment, the expression of the antiapoptotic protein Bcl‐2 decreased, while the levels of proapoptotic proteins Bax and cleaved Caspase 3 increased, confirming the superior anticancer efficacy of Lig@O‐P (Figure 4j).

**FIGURE 4:**
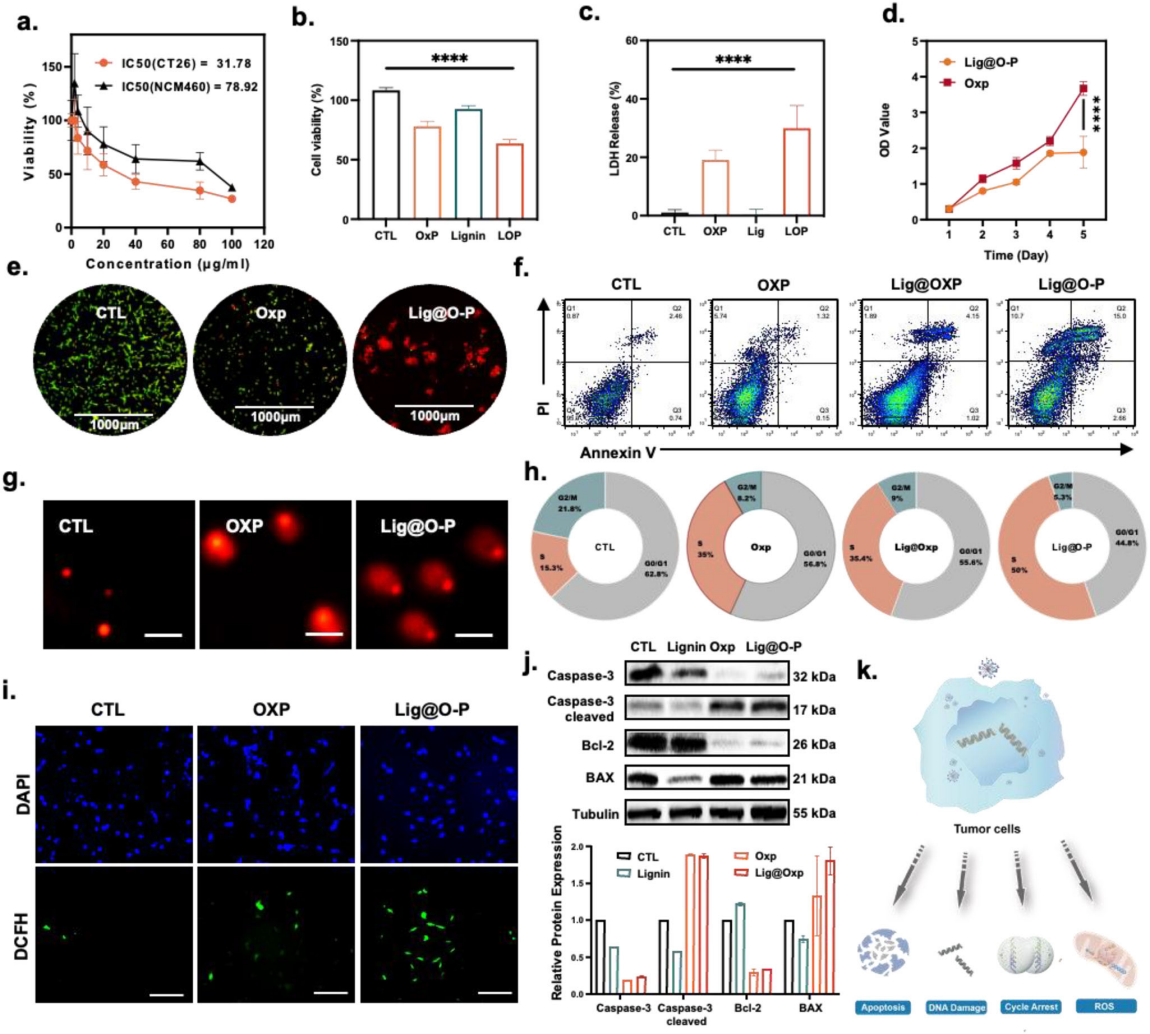
In vitro effect of Lig@O‐P on cancer cells. (a) Cytotoxicity tests after 48 h by treatment of Lig@O‐P in CT26, HCT116, and NCM460 cells. (b) Cell viability on CT26 cells after the indicated treatment for 48 h by Oxp, Lignin, Lig@O‐P (quantified by Oxp, 30 µg/ml). (c) LDH release on CT26 cells after 48 h of treatment with 30 µM Lig@O‐P. (d) Cell proliferation assay on CT26 cells after 48 h treatment with 30 µM Lig@O‐P in 5 days. (e) Live/dead assay by fluorescence microscope on CT26 cells after Lig@O‐P or Oxp (50 µg/ml, 48 h) treatment. (f) Apoptosis assay by FCM on CT26 cells after 24 h treatment by Oxp, Lig@OXP and Lig@O‐P. (g) Comet assay on CT26 cells after 38 h treatment of OXP and Lig@O‐P on 30 µg/ml of Oxp. (h) Cell cycle assay on CT26 cells after 24 h treatment with 30 µM Oxp, lignin, and Lig@O‐P. (i) Fluorescence microscopy image of ROS assay by DCFH probe on 30 µM Oxp and Lig@O‐P. Scale bar: 100 µm. (j) Western blotting of Casepase‐3, Bcl‐2, and Bax in CT26 cells after the indicated treatment by 30 µM Oxp, lignin, and Lig@O‐P for 24 h. (k) Illustration of the Lig@O‐P cell killing ability. LOP: Lig@Oxp‐αPD‐L1; LDH: lactate dehydrogenase; FCM: flow cytometry; DCFH: 2′,7′‐dichlorodihydrofluorescein diacetate. Data are represented as means ± SD (*n* > = 3) (*p < 0.05, ***p < 0.001, and ****p < 0.0001).

To further validate the toxic effects of Lig@O‐P on tumor cells, we performed a comet assay to evaluate DNA tailing post‐treatment (Figure 4g). As shown in Figure S15c, Lig@O‐P induced 54.4% of the mean tail moment, significantly higher than the control (0.5%) and oxaliplatin (6.7%) groups, indicating more pronounced DNA damage. FCM cell cycle analysis demonstrated a reduction in the percentage of cells in the G1 phase and an increase in the G2/M phase after Lig@O‐P treatment (Figures 4h and S15d). Intracellular ROS levels, measured by fluorescence microscopy and FCM using DCFH‐DA, were markedly elevated in Lig@O‐P‐treated cells, nearly doubling compared to the control group (CTL) (Figures 4i and S16a,b). DCFH‐DA is cleaved intracellularly by esterase and oxidized by ROS to produce green fluorescence, confirming the increased ROS levels. Western blot analysis of PARP (a DNA repair enzyme) and cyclin D1 (a cell cycle regulatory protein) also indicated DNA damage (reduced PARP levels and increased cleaved PARP levels) and cell cycle arrest (decreased cyclin D1 levels) (Figure S17). In summary, Lig@O‐P demonstrates significant cytotoxicity in CRC cells by inducing apoptosis, causing DNA damage, arresting the cell cycle, and increasing ROS production (Figure 4k).

### 2.5 Combination of Lig@O‐P and 5‐Fu Achieved a Synergistic FOLFOX Chemotherapy

In FOLFOX chemotherapy, the inhibition of TS and disruption of DNA metabolism are believed to drive the cytotoxicity of 5‐FU [19]. Forming stable complexes with TS, FnA effectively enhances the chemotherapeutic potency of FOLFOX [20]. To explore how modified lignin nanodrugs enhance FOLFOX chemotherapy, we first examined the ability of Lig@O‐P to substitute FnA and form stable complexes with TS. The binding mode of Arg‐GGE‐Me to TS was determined through autodocking (Figures 5a,b and S18). The docking showed that GGE binds with two backbone hydrogen bonds and a few van der Waals contacts, but no charged or π‐cation anchor. The guanidinium moiety of ARG in ARG‐GGE did not form a salt bridge. Instead, it wedged into a more hydrophobic pocket and donated extra hydrogen bonds to nearby polar side chains, tightening the fit of the lignin core. Furthermore, methylation of the guanidinium terminus in ARG‐GGE‐Me drove the lignin fragment into an optimal pose within the TS pocket, establishing a strong π‐cation contact (∼4.2 Å) with an aromatic side chain and reinforcing a bona fide salt‐bridge to a conserved carboxylate, while retaining additional hydrogen bonds and van der Waals contacts to yield an intermolecular energy of −11.4 kcal/mol. Although lignin is a high‑molecular‑weight polymer whose full binding dynamics extend beyond the confines of the pocket, these simulations reveal the core molecular drivers of affinity. It also suggests that for lignin, which tends to aggregate, these interactions facilitate more effective complex formation with TS, thereby inhibiting the normal catalytic function of the TS. Interestingly, when studying the interactions between lignin dimers and dUMP before and after modification using DFT (Figure S19), it was found that the interaction between amino‐acid‐modified lignin (ARG‐GGE) and dUMP doubled (−68.68 kcal/mol) compared to the unmodified lignin dimer (GGE). Furthermore, the interaction between methylated GGE lignin (ARG‐GGE‐Me) and dUMP was even stronger (−168.47 kcal/mol), indicating that the methylated lignin forms an extremely stable complex with dUMP. Collectively, these data suggest methylated ARG‐modified lignin may engage TS‐dUMP complexes, potentially disrupting dUMP→dTMP conversion via dual pathways.

**FIGURE 5:**
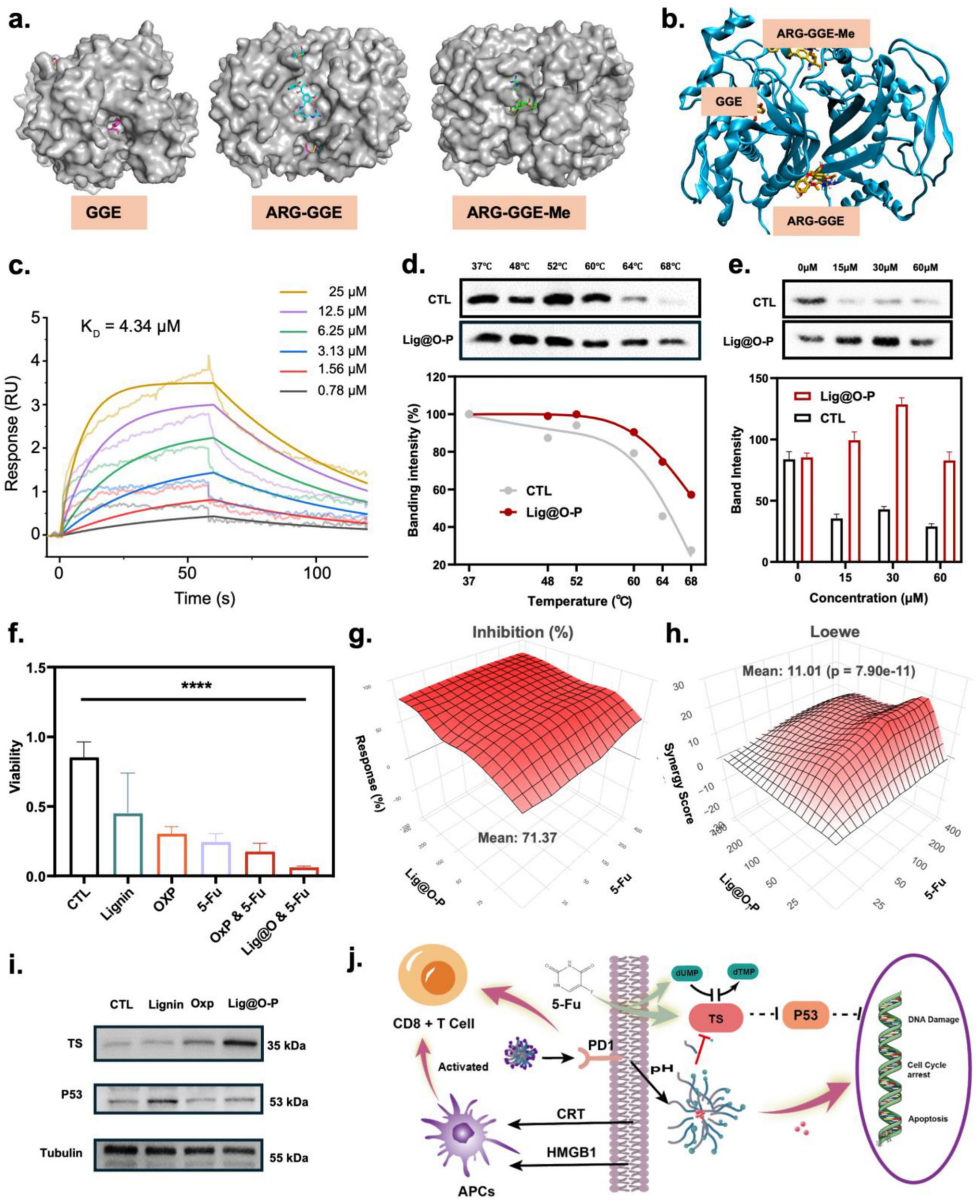
Combination application of Lig@Oxp and 5‐Fu components for cancer. (a, b) Binding of different modified‐lignin to TS active sites through molecular docking. (c) The SPR sensogram (response unit) representing the interaction between Arg‐EHL‐Me with recombinant TS protein. (d, e) Representative result of a cellular thermal shift assay (CETSA) at different temperatures (d) and concentrations (e). (f) Cytotoxicity tests after 48 h by different treatment in CT26; (g, h) automated calculation (g) and visualization (h) of Loewe synergy score analysis with Lig@O‐P and 5‐fluorouracil (5‐FU) at indicated doses to test for the synthetic lethality of compounds in CT26 cells. (i) Representative western blotting results of TS and P53 in CT26 cells after the indicated treatment by 30 µM Oxp, lignin, and Lig@O‐P for 24 h. (j) Proposed mechanistic model of the Lig@O‐P‐assisted FOLFOX‐like therapeutic strategy, including TS‐associated interaction of the released modified lignin, oxaliplatin release, and downstream immune activation. This schematic is intended as a conceptual working model based on the docking, SPR, CETSA, and biological data, rather than a direct structural representation of a fully resolved TS‐dUMP‐lignin complex. GGE: guaiacylglycerol‐beta‐guaiacyl ether, unmodified lignin; TS: thymidylate synthase; PD‐1: programmed cell death protein 1; CRT: calreticulin; HMGB1: high mobility group protein B1; APCs: antigen‐presenting cells. Data are represented as mean ± SD (*n* > = 3) (****p < 0.0001).

Molecular docking analysis was further examined to clarify the binding mode of the modified lignin toward TS. We have conducted a rigorous atomic‐level analysis of the docked complexes using the protein–ligand interaction profiler (PLIP) tool to uncover the precise binding domain and active sites of methylated ARG‐modified lignin (ARG‐GGE‐Me) on TS. As demonstrated by the detailed interaction profiler, the engineering of biological methylation mimicry grants the lignin derivative highly coordinated, specific multivalent recognition inside the TS active site cavity. Specifically, the aromatic group of the lignin core establishes a highly specialized π‐cation interaction with ARG‐163B at an exact distance of 4.21 Å, which drives the fragment into an optimal orientation within the active domain, while the newly introduced methylated guanidine group forms a robust salt bridge with the conserved acidic residue ASP‐174B at a distance of 5.22 Å to provide a strong electrostatic anchor. This structural placement is further locked by a densely coordinated hydrogen bonding network consisting of seven distinct bonds: ARG‐163B engages in dual side‐chain hydrogen bonds with the ligand's oxygen atoms at distances of 3.95 and 3.38 Å; THR‐167B and THR‐170B form three strong hydrogen bonds at 3.71, 3.17, and 3.41 Å to stabilize the polar interfaces; and ARG‐176B and ILE‐178B reinforce the network via close‐range hydrogen bonds at 3.05 and 2.68 Å, respectively. Additionally, the hydrophobic skeleton of the modified lignin perfectly matches the nonpolar sub‐pocket of TS, generating seven critical hydrophobic interactions with LEU‐121A (3.49 Å), dual PHE‐123A (3.85 and 3.38 Å), ARG‐163B (3.32 Å), PRO‐193A (3.99 Å), and dual contacts with LEU‐187A (3.06 and 3.00 Å). This alignment establishes a strong π‐cation contact (∼4.2 Å) with an aromatic side chain and reinforces a robust, bona fide salt bridge to a conserved carboxylate residue. Combined with retained hydrogen bonds and van der Waals forces, this modification yields an exceptionally stable final intermolecular energy of −11.4 kcal/mol. While lignin is a complex, high‐molecular‐weight polymer that extends past the immediate pocket boundaries, these computational findings perfectly clarify the core molecular drivers governing its TS affinity. This interaction successfully blocks the regular catalytic pocket of TS, mimicking the stabilizing action of FnA.

To directly assess the binding specificity between the modified lignin and TS, we performed surface plasmon resonance (SPR) analysis (Figure 5c). Recombinant human TS was immobilized on a sensor chip, and the AA‐methylated lignin (HIS‐EHL‐ME) was injected as the analyte. The interaction exhibited clear concentration‐dependent binding, yielding an equilibrium dissociation constant (KD) of 4.34 µM based on kinetic fitting, with a steady‐state affinity of 16.2 µM. This low‐micromolar range affinity confirms a direct and specific molecular interaction between the modified lignin polymer and the TS protein. To verify our simulation, cellular thermal shift assay (CETSA) was employed and revealed that CT26 lysates treated with Lig@O‐P exhibited an increase in TS melting temperature from 37°C to 68°C (Figure 5d), suggesting a direct interaction between Lig@O‐P and TS. This interaction was further validated by an isothermal dose‐response fingerprint CETSA (ITDRF‐CETSA), which showed that Lig@O‐P dose‐dependently (from 0 to 60 µM) bound to and stabilized the TS protein (Figure 5e). Thus, Lig@O‐P functions as a direct binding partner of TS, forming stable complexes similar to FnA. To further assess whether CRC cells were more susceptible to Lig@Oxp‐based FOLFOX, CT26 cells were treated with various combinations, including PBS (control), lignin, oxaliplatin (Oxp), 5‐FU, traditional FOLFOX (Oxp combined with 5‐FU), and Lig@O‐P‐based FOLFOX (Lig@O‐P combined with 5‐FU). The results indicated that Lig@O‐P combined with 5‐FU exhibited greater cytotoxicity against CRC cells than other groups (Figure 5f). Drug concentrations ranging from 0 to 400 µmol/L of Lig@O‐P and 5‐FU were tested to measure their effects on cell survival, generating a concentration‐response matrix where 100% corresponds to complete cell death, and 0% indicates no difference in survival (Figure 5g). Synergy between the two drugs was evaluated using SynergyFinder 2.0 [21]. The results demonstrated effective synergy scores for the combination (Figure 5h), with a Loewe synergy score of 11.01, Bliss Loewe score of 4.498, HSA score of 16.476, Bliss score of 9.217, and Loewe score of 6.649 (Figure S20). These data confirm that Lig@O‐P and 5‐FU exhibit a significant synergistic effect against CRC cells. Additionally, 5‐FU‐induced nucleolar stress results in the release of ribosomal proteins, activating the P53 pathway [22]. Western blotting analysis revealed that treatment with Lig@O‐P + 5‐FU led to a marked increase in TS protein levels, which is a recognized consequence of effective TS functional inhibition and the disruption of its autoregulatory translational feedback loop. Concomitantly, P53 expression was reduced (Figure 5i). This result indicates that Lig@O‐P, in combination with 5‐FU, potently suppresses TS activity, as evidenced by the characteristic compensatory rise in TS protein. This effective inhibition contributes to the anti‐cancer efficacy, alongside the observed modulation of the P53 pathway.

Furthermore, recent studies indicate that oxaliplatin can induce a form of apoptosis known as immunogenic cell death (ICD), which triggers immune responses by activating T lymphocytes to recognize tumor‐specific antigens [23]. ICD inducers activate damage‐associated molecular patterns (DAMPs), including the exposure of calreticulin (CRT) and the translocation of high mobility group protein B1 (HMGB1) [24]. In this study, the potential of lignin‐based FOLFOX for ICD‐induced immunogenicity was evaluated by assessing CRT exposure and HMGB1 release. Lig@O‐P‐treated CT26 cells showed significantly expression of CRT and HMGB1 (Figure S21), indicating that ICD was induced in CRC cells after Lig@O‐P treatment. Therefore, Lig@O‐P binds to PD‐L1 on tumors and enters cells via the lysosomal pathway, where it disintegrates in the acidic lysosomal environment, releasing modified lignin and oxaliplatin. The release of Arg‐EHL‐Me forms a stable complex with the TS and dUMP, enhancing 5‐FU's ability to damage dUMP and regulate P53 expression. The released oxaliplatin synergizes with 5‐FU and Arg‐EHL‐Me in FOLFOX chemotherapy, resulting in DNA damage, cell cycle arrest, and apoptosis. Additionally, Lig@O‐P treatment promotes the release of ICD‐related molecules such as CRT and HMGB1 and antagonizes PD‐L1 interaction, alleviating T cell immunosuppression and further enhancing immune activation (Figure 5j). Thus, Lig@O‐P augments the tumor‐killing efficacy of the FOLFOX regimen by inactivating TS through complex stabilization while synergizing with immune activation.

### 2.6 In Vivo Anticancer Efficacy of Lig@O‐P

The in vivo anticancer efficacy of Lig@O‐P was assessed in BALB/c mice bearing CT26 tumors. The mice were randomly divided into four groups (*n* = 4 per group) and intravenously injected five times on Days 1, 4, 7, 10, and 13 with PBS (control), lignin (empty nanoparticles), oxaliplatin (free drug), or Lig@O‐P, each at a dose equivalent to 5 mg/kg of oxaliplatin (Figure 6a). As shown in Figure 6b, tumors in the PBS and lignin‐treated mice grew rapidly. In contrast, oxaliplatin treatment significantly inhibited tumor growth, and Lig@O‐P treatment resulted in even greater tumor suppression. The antitumor efficacy of the various treatments was further confirmed by tumor photographs (Figure 6c) and the weights of the excised tumors after 14 days of treatment (Figure 6d), both of which corroborated the superior antitumor effect of Lig@O‐P. Additionally, the oxaliplatin‐treated group exhibited significant weight loss (Figure 6e), indicating severe systemic toxicity associated with the free drug. In contrast, the Lig@O‐P group showed no significant weight changes, consistent with previous studies suggesting that lignin nanoparticles can mitigate the toxicity of chemotherapeutic agents [25].

**FIGURE 6:**
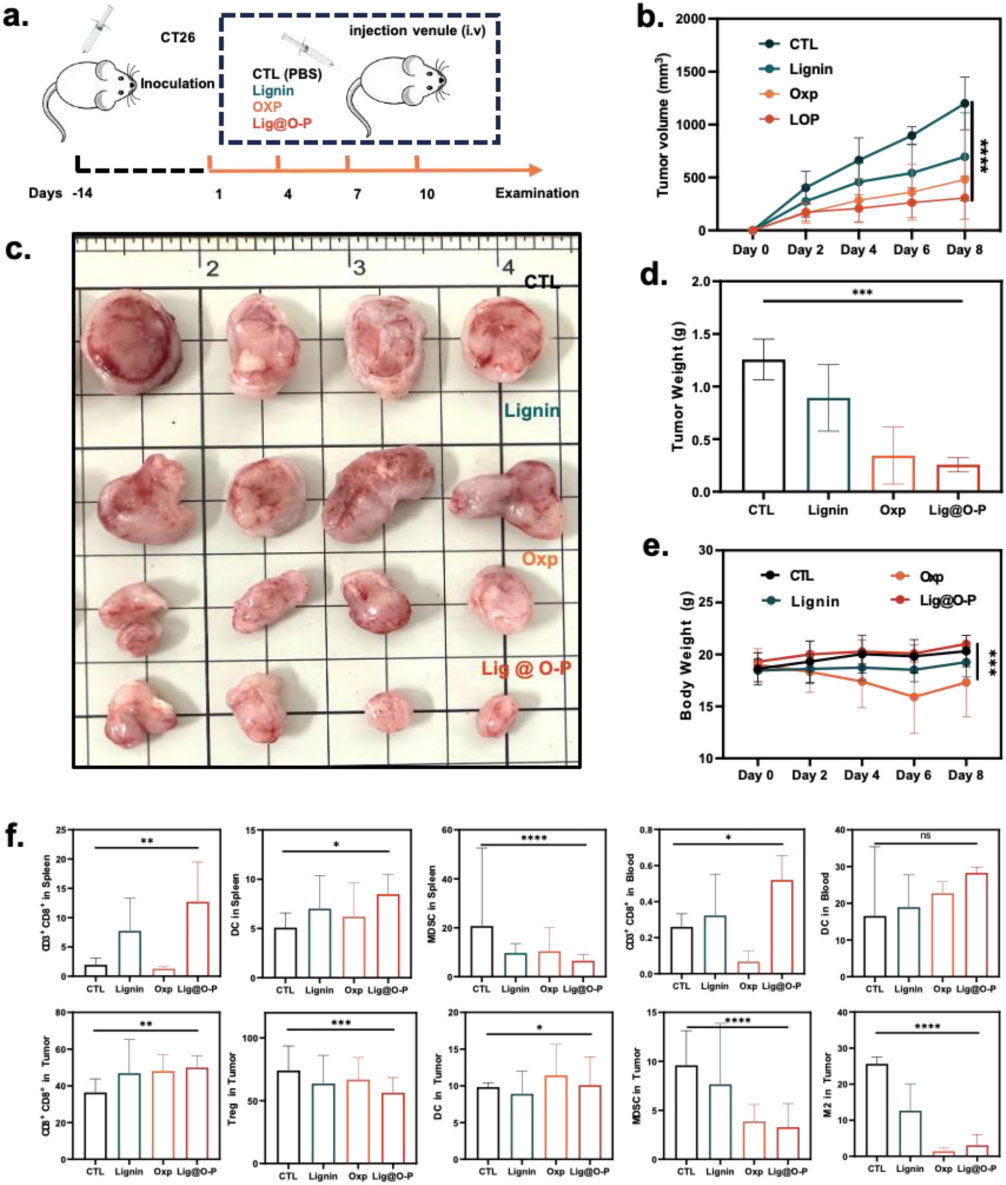
Lig@O‐P (LOP) inhibits the progression of CRC in vivo. (a) Illustration of the experiment design and treatment schedule (*n* = 5 per group); (b) Tumor volume (percentage of D0) during the treatment in Day 8; (c) Digital pictures of tumors from the CT26 tumor‐bearing mice after 8 days of therapy; (d) Average tumor weight excised from the CT26 tumor‐bearing mice after 8 days of treatment; (e) Body weight change during the treatment in Day 8; (f) Level of CD3+ T cells, CD4+ T cells, memory CD8+ T cells, Treg cells, Macrophages cells, M2 cells, activated DCs, and MDSCs in tumors on Day 18, analyzed by flow cytometry. DCs: dendritic cells; MDSCs: myeloid‐derived suppressor cells; CRC: colorectal cancer; *n*  =  4, ** *p* < 0.01, *** *p* < 0.001, ****p < 0.0001; NS  =  no significance.

Our previous results demonstrated that ICD contributes to the therapeutic effect of Lig@O‐P (Figure S21). Given that oxaliplatin is a well‐known ICD inducer and Lig@O‐P antagonizes the immune checkpoint inhibitor PD‐L1, we hypothesized that Lig@O‐P could prime anticancer immune responses. To test this hypothesis, we performed FCM to evaluate immune cell infiltration in blood, spleen, and tumor tissues. As shown in Figure 6f, CD8+ T lymphocyte levels were markedly increased in tumors, blood, and spleen following Lig@O‐P treatment, accompanied by the activation of cytotoxic T lymphocytes. Additionally, CD86+ dendritic cells (DCs) were significantly activated in the tumor, spleen, and blood by Lig@O‐P. Corresponding to these immune‐stimulatory effects, there was a significant reduction in the levels of myeloid‐derived suppressor cells (MDSCs) in the spleen and tumor, regulatory T cells (Tregs) in the tumor, and tumor‐associated macrophages (M2) in the tumor. Collectively, these findings indicate that Lig@O‐P effectively inhibits tumor growth in vivo and enhances immune activation, further promoting the destruction of cancer cells.

### 2.7 Combination of Lig@O‐P and 5‐Fu Achieved FOLFOX Chemotherapy in CRC Mice

The combination therapy of Lig@O‐P and 5‐FU for FOLFOX chemotherapy was further evaluated in vivo using CT26‐derived CRC models (Figure 7a). A dosage of 10 mg/kg of 5‐FU was selected for the in vivo combination treatment. The mice were randomly divided into four groups (*n* = 5 per group) and intravenously injected five times on Days 1, 4, 7, 10, and 13 with PBS (control), oxaliplatin, 5‐FU (free drugs), Oxp & 5‐FU (classical FOLFOX), or Lig@O‐P & 5‐FU (lignin‐based FOLFOX). As shown in Figure 7b, tumors grew rapidly in the mice treated with PBS and lignin. In contrast, both classical FOLFOX and lignin‐based FOLFOX treatments significantly inhibited tumor growth. Notably, in the lignin‐based FOLFOX group, tumors in two mice nearly regressed completely, demonstrating the superior efficacy of the combined FOLFOX regimen. Antitumor efficacy was further confirmed through tumor photographs (Figure 7c) and the weights of the excised tumors (Figure 7d), which consistently demonstrated that the Lig@O‐P combined FOLFOX regimen was more effective in suppressing colorectal tumor growth. Body weight data (Figure 7e) also showed that the Lig@O‐P combined FOLFOX treatment effectively reduced the toxicity of the chemotherapeutic drugs. Additionally, excised tumors were fixed and analyzed by immunohistochemistry (IHC) (Figure 7f). The expression of Ki‐67 was significantly attenuated in tumors from the lignin‐based FOLFOX treatment group compared to the other groups. Positive staining for CRT and HMGB1 was also significantly upregulated in the lignin‐combined FOLFOX‐treated mice, consistent with previous studies and further indicating immune system activation. Moreover, TS expression was significantly upregulated in the Lig@O‐P & 5‐FU group, consistent with in vitro results (Figure 5g), further supporting the idea that the Lig@O‐P‐based FOLFOX regimen enhances chemotherapy efficacy by modulating TS. In conclusion, the Lig@O‐P‐based FOLFOX chemotherapy regimen demonstrated a superior capacity for treating CRC.

**FIGURE 7:**
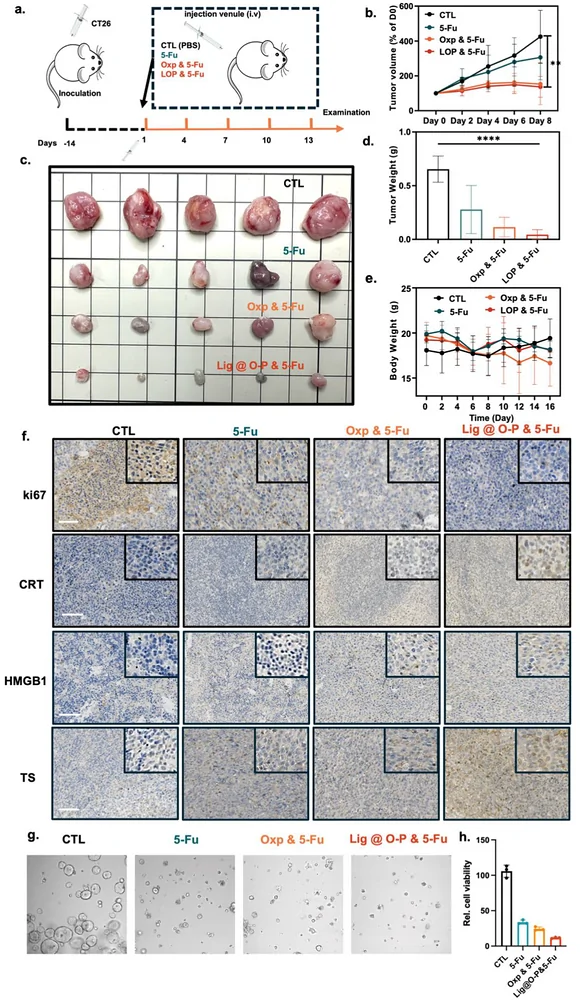
The combination therapy of Lig@O‐P and 5‐Fu for CRC mouse model. (a) Illustration of the experiment design and treatment schedule (*n* = 5 per group). (b) Tumor volume (percentage of D0) during the treatment in Day 14. (c) Digital pictures of tumors from the CT26 tumor‐bearing mice after 14 days of therapy. (d) Average tumor weight excised from the CT26 tumor‐bearing mice after 14 days of treatment. (e) Body weight change during the treatment in Day 16. (f) Level of ki67, CRT, HMGB1, and TS in tumors analyzed by immunohistochemistry (IHC) (g) Representative bright‐field images of CRC‐PDOs subjected to four sequential treatments with PBS, free 5‐FU, oxaliplatin + 5‐FU (classical FOLFOX), or Lig@O‐P + 5‐FU, with images acquired over 3 days. (h) ATP‐based viability analysis of CRC‐PDOs after 3 days of treatment. (** *p* < 0.01, *** *p* < 0.001, ****p < 0.0001; NS  =  no significance).

To further strengthen the translational relevance of the Lig@O‐P‐based FOLFOX strategy, we additionally evaluated its therapeutic effect in patient‐derived colorectal cancer organoids (CRC‐PDOs), which are recognized as a clinically relevant ex vivo model that preserves key features of patient tumors, including heterogeneity and chemotherapy‐response characteristics. As shown in Figures 7g,h and S23, CRC‐PDOs were subjected to treatments with PBS, free 5‐FU, oxaliplatin + 5‐FU (classical FOLFOX), or Lig@O‐P + 5‐FU, and organoid morphology was monitored every 12 h. Over time, PDOs in the PBS group grew rapidly, whereas both the classical FOLFOX group and the Lig@O‐P + 5‐FU group markedly suppressed organoid growth by Day 3. Consistently, ATP‐based viability analysis after 3 days of treatment further confirmed the inhibitory effect of the combination regimens on CRC‐PDO growth. These results provide additional human patient‐derived evidence supporting the translational potential of the Lig@O‐P‐based FOLFOX regimen.

### 2.8 In Vivo Toxicity Studies

To evaluate the in vivo application of Lig@O‐P, a hemolysis test was conducted to assess its biosafety. No significant hemolysis was observed for Lig@O‐P at concentrations up to 1 mg/mL compared to the PBS negative control (Figure 8a), indicating the nanocomposite's high blood compatibility. Serum liver and kidney function markers were also analyzed to further evaluate systemic toxicity (Figure 8b–e). The levels of aspartate aminotransferase (AST), alanine aminotransferase (ALT), lactate dehydrogenase (LDH), and blood urea nitrogen (BUN) in the therapeutic groups showed no significant elevation compared to the PBS control (Figure 8b–e). However, BUN levels in the free oxaliplatin group were significantly increased, with a trend toward elevated AST and ALT levels (Figure 8b,c), though these changes were not statistically significant. These findings suggest that free oxaliplatin induces systemic toxicity, consistent with previous reports. Notably, the Lig@O‐P group demonstrated normal levels of these biomarkers. Histopathological analysis using H&E staining further confirmed the absence of systemic toxicity (Figure 8f). No significant tissue damage was observed in the PBS and Lig@O‐P groups. These results collectively indicate that Lig@O‐P does not cause systemic toxicity. Additionally, in line with our previous hypothesis (Figure 6e), lignin effectively reduces the toxicity of chemotherapeutic agents, particularly in the kidneys.

**FIGURE 8:**
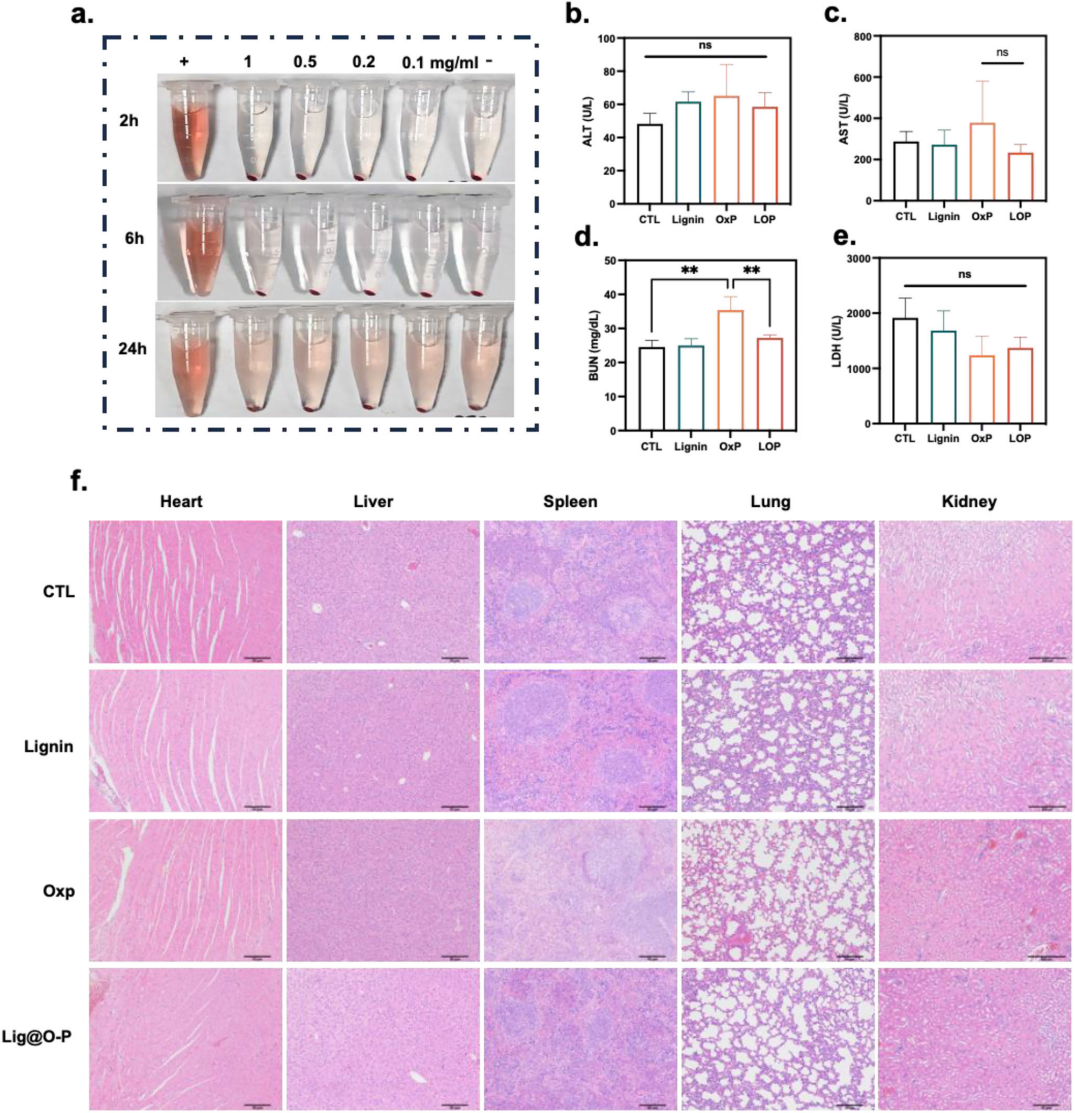
Blood and organ toxicity of the Lig@O‐P nanocomposite in vivo. (a) Hemolysis experiments in varying concentrations (0.1–1 mg/mL) of Lig@O‐P compared to positive (H_2_O) and negative (PBS) controls after 4 h incubation. (b–e) Serum biochemical markers of organ function after final treatment: (b) ALT and (c) AST for hepatic injury assessment; (d) BUN for renal function; (e) LDH for cellular damage (*n* = 3). (f) Histopathological analysis of major organs (heart, liver, spleen, lung, and kidney) via hematoxylin and eosin (H&E) staining (scale bar: 50 µm), showing no significant morphological alterations. ALT: alanine aminotransferase; AST: aspartate transaminase; BUN: blood urea nitrogen; LDH: lactate dehydrogenase.

## 3 Discussion

Recently, there has been increasing interest in leveraging the redox polyphenol chemistry of lignin to develop novel biomedical materials for drug delivery systems [18, 26]. However, due to the inherent complexity of lignin and the heterogeneity introduced by different extraction methods, its biological applications remain limited. In this study, we developed a methylated, AA‐grafted lignin inspired by protein methylation (Figure 1a), which exhibited a strong positive charge after ARG modification (Figure 1c) and demonstrated pH‐responsive charge variations (Figure S5). We then optimized the synthesis process to form highly uniform, strongly electropositive, and pH‐sensitive nanoparticles (Figure 1d). This approach can be applied to various lignin structures, broadening lignin's potential applications in nanomaterial‐based biomedical practices.

Importantly, the “protein methylation‐inspired” nature of our design should be understood in a physicochemical rather than biochemical sense. In proteins, lysine/ARG methylation does not simply switch formal charge, but can reshape local electronic distribution, steric environment, hydrophobicity, and interaction patterns, thereby modulating biomolecular recognition by partner proteins and methylation‐reader domains [27, 28]. Accordingly, our amino‐acid grafting and subsequent methylation of lignin do not aim to reproduce enzymatic post‐translational methylation itself, but rather to emulate some of its interfacial consequences. Consistent with this view, the DFT‐derived ESP maps in Figure 1b show broadened electrostatic distribution and enriched local positive potential after methylation, while the TS/dUMP interaction data support a concomitant change in binding propensity. Therefore, the biomimetic value of this platform lies in methylation‐enabled regulation of interfacial electrostatics and molecular interactions.

Charge‐reversal nano‐systems have garnered increasing attention recently due to their unique properties [29]. These systems are neutral or negatively charged under physiological conditions but switch to a positive charge in the tumor or disease environment [30, 31, 32]. This charge reversal enhances their stability in the bloodstream and increases cellular uptake at the tumor site, making them excellent candidates for targeted drug delivery with enhanced biosafety [33, 34]. In this study, we developed a modified lignin‐based charge‐reversal targeting nanocarrier (Figure 2a). The nanocarrier demonstrated significant charge‐reversal ability (Figure 2f), uniform nanoparticle size (Figure 2b–e), and exceptional stability in physiological conditions (Figure 2h). We also evaluated its drug‐loading and release capabilities (Figure 2i), confirming its suitability for use as a drug delivery system. These results highlight the feasibility of utilizing modified lignin in biological applications. Moreover, we designed a novel nanocarrier that incorporates both charge‐reversal and targeted antibody pathways, opening up possibilities for further optimized drug delivery.

FOLFOX, a classical chemotherapy regimen, has been a mainstay in the treatment of advanced cancer for decades [10]. However, its limited curative effect, high side‐effect profile, and long treatment duration continue to pose challenges for both clinicians and patients [4, 5]. Nano‐FOLFOX systems have been shown to address these limitations by prolonging in vivo circulation times and enhancing the therapeutic efficacy of FOLFOX [35]. In our research, we created a charge‐reversal modified lignin carrier to deliver FOLFOX, named Lig@O‐P. Lig@O‐P exhibited effective targeted delivery both in vitro and in vivo (Figure 3a–h). Our studies demonstrated that Lig@O‐P showed marked antitumor activity against CRC through mechanisms such as apoptosis, DNA damage, cell cycle arrest, and ROS production (Figure 4i). Lig@O‐P also showed significant in vivo antitumor activity against CRC (Figure 6b–e). Furthermore, the combination of Lig@O‐P and 5‐FU achieved an impressive therapeutic effect in FOLFOX chemotherapy for CRC (Figures 5, 6, 7, 8 and Figures 7, 8). Notably, Lig@O‐P not only demonstrated low biological toxicity but also significantly reduced the renal toxicity associated with chemotherapy (Figures 6e and 8a–f), consistent with findings from previous studies [25]. Overall, the novel Lig@O‐P‐based FOLFOX delivery system showed enhanced therapeutic efficacy and reduced chemotherapy‐associated renal toxicity.

TS is a key protein that enhances the response to 5‐FU via FnA in the FOLFOX regimen [19, 36]. Importantly, we found that modified lignin can mimic the action of FnA by forming classical complexes with TS. This may explain why Lig@O‐P enhances the efficacy of FOLFOX chemotherapy and represents a significant discovery regarding lignin's biological functions (Figure 5a–f). Additionally, oxaliplatin has been shown to shift the tumor microenvironment from “cold” to “hot” through ICD. Studies have demonstrated that combining nano‐FOLFOX with anti‐PD‐L1 antibodies can effectively enhance antitumor outcomes [35]. In our study, we introduced a PD‐L1 antibody to create targeted nanoparticles. The results indicated that ICD occurred following Lig@O‐P treatment (Figures 6f and S21), suggesting that this mechanism could contribute to the enhanced efficacy of Lig@O‐P‐based FOLFOX. Thus, we have not only developed a safer and more effective nanomaterial but also provided valuable insights into its underlying mechanism.

Our formulation offers a new application strategy for lignin materials, improving their biological properties and providing a novel FOLFOX delivery system that brings us closer to clinical translation. However, several areas require further exploration:

1. Although methylation plays a critical role in biological processes, our material design was inspired by protein methylation, which imparts unique bioactivity to lignin, such as its ability to bind TS and dUMP. Atomic‐level analysis reveals that methylation induces an electron redistribution in lignin, similar to protein methylation, resulting in significant changes in its ESP distribution and thereby generating a range of bioactivities. However, is methylation the only modification capable of producing such effects? Could other modifications that regulate ESP, such as acetylation, confer different bioactivities to lignin? We believe these ideas could unlock even more potential in lignin's functional capabilities.
2. We utilized three AAs to modify lignin and obtain a strongly positive lignin variant, but this method can theoretically be applied to other AAs. Could modifying lignin with other functional AAs, such as cysteine, selenocysteine, or methionine, allow lignin to adopt additional biological functions for various applications?
3. While this study establishes the efficacy of methoxy‐enriched EHL as a biomimetic nanocarrier, future work will systematically investigate the influence of lignin source, molecular weight, and precise methoxy group content on chemosensitization efficacy. Furthermore, comparative studies evaluating other natural or synthetic methoxy‐rich biomolecules for similar applications would provide deeper insights into the specificity and generalizability of the “methylation mimicry” strategy.
4. Although we demonstrated that lignin could replace FnA in forming classical complexes with TS, how does this compare to leucovorin in terms of complex formation and stability? What differences exist, and could lignin completely replace FnA in clinical practice? These questions require further investigation.
5. To better understand the clinical translation potential of this nanomaterial, the performance of modified lignin in human biological environments needs to be further elucidated. For instance, what happens to positively be charged lignin during blood circulation after antibody blocking? Is there a formation of a protein corona that affects its targeting ability? Additionally, more mammalian or patient‐derived xenograft (PDX) models should be employed to further evaluate this nanomaterial.
6. In both this and previous studies, we found that lignin can alleviate the renal toxicity of chemotherapy drugs (e.g., doxorubicin and oxaliplatin). This is an exciting finding, suggesting that lignin may have therapeutic potential for treating kidney injury‐related diseases, warranting further exploration of lignin's broader biological value.

The mechanism underlying this renoprotective effect should, however, be interpreted cautiously. In the present study, our evidence primarily supports a protective phenotype, rather than a kidney‐specific mechanistic demonstration. Mechanistically, this effect may arise from two non‐mutually exclusive factors. First, nanoparticle‐mediated delivery may reduce off‐target renal exposure to free oxaliplatin. Second, the lignin component itself may contribute because lignin is a polyphenolic material with redox‐active phenolic groups. Such properties may help attenuate chemotherapy‐associated oxidative stress and the secondary inflammatory injury in renal tissue.

## 4 Conclusion

In this study, we developed an AA methylation‐modified lignin to create a pH‐responsive charge‐reversal nano system. This modified lignin enabled targeted delivery of FOLFOX nanodrugs, significantly enhancing both the therapeutic efficacy and biosafety of CRC treatment. Additionally, we confirmed that the modified lignin could form stable complexes with TS, further contributing to its therapeutic potential. Overall, we propose a novel lignin‐based FOLFOX nanomedicine, offering a new first‐line chemotherapy drug delivery strategy that improves treatment outcomes and reduces side effects. This work not only introduces an innovative lignin‐based material but also expands the potential applications of lignin in biological systems, providing new insights into the use of complex macromolecular polymers in biomedicine.

## 5 Method

### 5.1 Materials

EHL was sourced through the LignoBoost process in Finland. Formaldehyde solution (HCHO, 37%), TMP, dialysis membranes, and AAs, including ARG, glutamate (GLU), and GLY were obtained from Aladdin (Shanghai, China). Sodium hydroxide (NaOH) was bought from Guangzhou Chemical Reagent Factory (Guangzhou, China). Oxaliplatin, 5‐Fu was purchased from Selleck. The anti‐mouse PD‐L1, anti‐mouse ki67, anti‐mouse CRT, anti‐mouse HMGB1, anti‐mouse TS, and anti‐mouse p53 were purchased from Biolegend and the sources of antibodies used in the immunity assay were purchased from Biolegend.

### 5.2 Preparation of EHL‐Arg‐Lignin

The Mannich reaction along with the modified methylation reaction was performed based on previous work [12], Briefly, the reaction was divided into two parts. First, to produce the AA‐modified lignin, the Mannich reaction is employed to graft the EHL with AAs (Glu, Gln, and Arg) via a C─C bond. Specifically, 1 g of EHL and 6.8 mmol of AAs were dissolved in 30 mL of 0.1 M NaOH Solution. Then, the 37% formaldehyde aqueous solution (1.5 mL) was diluted with 10 mL of deionized water and slowly added to the mixture. The mixture is then incubated at 90°C for 4 h. The resulted lignin modified with different AAs was named at Me‐AL‐GLU, Me‐AL‐GLY, and Me‐AL‐ARG, respectively. During the subsequent methylation process, the solution of AA‐modified lignin was directly combined with varying amounts of TMP, which acted both as a solvent and a methylation agent. NaOH was then added, and the mixture was stirred, gradually increasing the temperature to 150°C over 30 min and maintaining it for 1 h. After the reaction, most of the TMP was removed via rotary evaporation. The methylated solutions of the three types of EHL‐AA were slowly dripped into water and dialyzed in 3500 Da dialysis bags for 2 days to eliminate excess unreacted reagents, resulting in solid powder upon freeze‐drying. The methylated EHL‐AA (Me‐EHL‐AA) with different AAs was designated as Me‐EHL‐GLU, Me‐EHL‐GLY, and Me‐EHL‐ARG, respectively.

To confirm the characterization of the materials, the size distribution and zeta potential of Me‐EHL‐AA nanoparticles were measured using a Zetasizer Nano ZS instrument (Malvern Instruments Ltd., UK). A SU8220 field emission scanning electron microscope (Thermo Fisher Scientific, Japan) was used to capture the high‐resolution scanning electron microscopy (HR‐SEM) images of the dried nanoparticles and the two‐dimensional heteronuclear single‐quantum coherence (2D‐HSQC) was used to investigate the composition and interunit linkage contents of lignin samples. Twenty‐five milligrams lignin samples were dissolved in 0.5 mL dimethyl sulfoxide‐d6 (DMSO‐d6). The NMR spectra were recorded on a 600 MHz NMR spectrometer (AVANCE III HD, Bruker, Germany).

GPC/SEC Analysis of Lignin. Prior to GPC/SEC analysis, lignin samples were acetylated to improve their solubility in THF. Briefly, 100 mg of lignin was dissolved in acetic anhydride (1 mL) and pyridine (1 mL) and reacted in the dark for 72 h. The reaction mixture was then poured into 40 mL of aqueous solution containing 0.33 mL concentrated hydrochloric acid to precipitate the acetylated lignin. The product was filtered, washed with deionized water, and dried. The acetylated lignin was dissolved in THF at 2 mg mL^−1^ and filtered through a 0.45 µm membrane. GPC/SEC analysis was performed on an Agilent 1260 system equipped with a differential refractive index detector and a PLgel Mixed‐C column at 30°C, using chromatographic‐grade THF as the mobile phase at a flow rate of 1.0 mL min^−1^. Mn, Mw, and dispersity (*Đ*) were calculated from the chromatographic data.

### 5.3 Preparation of Lignin@Oxp‐PD‐L1 (Lig@O‐P)

The supported drug nanoparticles were synthesized by the solvent exchanged method [37]. First, 10 mg of the freeze‐dried Me‐EHL‐AA and 1 mg of oxaliplatin were dissolved in a 1 mL 70%THF/water solution. This solution was dropped slowly into a 10 mL deionized (DI) water and stirred overnight to removal the organic solvent, resulting in a 10 mL solution of the Lignin@Oxp. Then, the solution was concentrated to 1 mL by ultrafiltration (3500 kDa, 3000 g, 10 min) to remove unreacted reagents and facilitate the antibody bonding. Next, 10 µl of the α‐PD‐L1 was added into the solution and reacted at room temperature to bind by electrostatic self‐assembly. Subsequently, the product is centrifuged for 30 min at 100000 g to remove the unreacted solvent again and washed twice using DI water twice to form the product Lig@O‐P. The nanoparticles were saved in 1 mL PBS at 4°C.

We used Zetasizer Nano ZS (Malvern Instruments Ltd., UK) to measure the hydrodynamic particle sizes, zeta potentials, and the stable of the Lig@O‐P at 25°C. The hydrodynamic particle sizes of the LNPs were determined from the measured diffusion coefficients using the Stokes–Einstein equation. The zeta potentials were calculated based on the measured electrophoretic mobility of the LNPs. The stable of the Lig@O‐P was stored in PBS for 28 days. The morphology of the Lig@O‐P was measured by the transmission electron microscope (TECNAI G2, FEI, Maastricht, the Netherlands) and the field emission scanning electron microscope (Ultra‐55, ZEISS, Oberkochen, Germany). Samples were mixed with potassium bromide after drying for FT‐IR measurements, the scan was performed from 4000 to 600 cm^−1^ after background subtraction (Thermo Fisher IS50R). Pt contents in Lig@O‐P were measured in the ICP‐MS (Agilent 7700).

### 5.4 The Drug Target and Biodistribution

The CT26, HCT116, and NCM460 is obtained from ATCC. These cells were maintained within DMEM (Gibco) with 10% bovine calf serum (Gibco), 1% antibiotic‐antimycotic (Gibco), and 1 µg/mL puromycin (ThermoFisher). Cells were maintained at 37°C with 5% CO2 and 95% relative humidity.

Cellular uptake of Lig@O‐P in vitro was evaluated in the PD‐L1 overexpressing CRC cell lines CT26 using FCM, FL microscopy, ICP‐MS, and bio‐TEM. Cells at designated densities (FCM and ICP‐MS, TEM, 1 × 106 cells/well; FL microscopy, 5 × 104 cells/well) were incubated in a 6‐well plate for 24 h and were then incubated with 30 µg/ml nanoparticles. For fluorescence loading, the nanoparticles used in FCM and FL microscopy were co‐incubation with Rhodamine‐B. After incubating 1, 4, and 6 h, Cells were washed with PBS three times and the FL intensity of Lig@O‐P was determined using FCM (SONY SA3800 Spectral FCM, JAPAN) and FL microscopy (olympus/FV300). For FL microscopy, the nuclei and lysosomes of cells on cover slides were stained with Hoechst 33342 and LysoTracker Green DND‐26, respectively, before observation using FL microscopy. For TEM assay, the cells were collated into 1.5 mL tubes and fixed in 2.5% glutaraldehyde overnight at 4°C. Then, the samples were prepared according to the published protocol and shoot by transmission electron microscope (RuliTEM, HT7800). Pt contents in oxaliplatin and Lig@O‐P treated cells were measured by ICP‐MS (Agilent 7700). For pH‐dependent antibody‐retention analysis, fluorescence‐labeled anti‐PD‐L1‐coated Lig@O‐P was incubated at pH 7.4 or pH 5.5 for 24 h, centrifuged at 30,000 rpm for 30 min, and the fluorescence of the nanoparticle‐associated fraction was comparatively analyzed.

All mice experiments were approved by the Animal Center of Sun Yat‐sen University (2023343) and complied with the regulations on the Administration of Laboratory Animals of the State Council of the People's Republic of China. 1 × 10^6^ CT26 cells in 100 µL of PBS were subcutaneously injected into BALB/c mice. 0.05 wt % of ICG was used to formulate ICG‐containing Lig@O‐P and Lig@Oxp. When the tumor volume reached about 100 mm^3^, nanoparticles were injected through the tail vein. Mice were observed at various time points (1, 4, 6, 8, 12, 24, 48, and 72 h) using NEOImage FOBI. Mice were sacrificed 48 h after injection, and tumor tissues were harvested. Organs (heart, liver, spleen, lung, kidney, and intestine) and tumors were then imaged for fluorescence.

### 5.5 In Vitro Cytotoxicity Assay of Lig@O‐P

For cytotoxicity assay, Colon cancer cells (CT26, HCT116) and normal cells (NCM460) were seeded in 96‐well plates at a density of 5 × 10^3^ cells per well and allowed to adhere for 24 h. Cells were then treated with different concentrations of free OxPt, Lignin, Lig@Oxp and Lig@O‐P, or 5‐Fu and Lig@O‐P add 5‐Fu for another 48 h. Cell viability was then detected using CCK8 assay (Promega, Madison, WI) with a scanning multiwell Spectrometer reader (Multiskan GO, Thermo Fisher Scientific), and the combine cytoxicity assay was performed by SynergyFinder. The LDH release assay was performed by LDH assay kit (Beyotime, C0016) and determined by spectrometer reader. For live/dead assay, CT26 cells seeded in 6‐well plate, and treated with PBS, OxP, and Lig@Oxp‐PD‐L1 (30 µg/mL), respectively. After 6‐h treatment, the cells further 1‐h incubation for Calcein AM‐PI live–dead cell staining. Afterwards, the cells were washed three times with PBS and subjected to confocal microscope imaging.

The comet assay was performed by comet assay kit (Beyotime, C2041M), and for the apoptosis assay, CT26 cells were seeded in six‐well plates (5 ×10^4^ cells/well) for 12 h to adhere. And then treated with OxPt, Lignin, and LNP for another 24 h. The cells were harvested, washed twice with ice‐cold PBS, stained with Alexa Fluor 488‐Annexin V and PI for 15 min at room temperature in the dark, and then analyzed by FCM (BD, Bioscience). For cell cycle assay, treated CT26 cells as described above were collected, washed twice with ice‐cold PBS, fixed with 70% ethanol at 4°C overnight, and treated with RNase A for 45 min, and followed by PI staining for 30 min. The alteration of cell cycle was analyzed by FCM. The DCFH‐DA assay kit was used for ROS detection. CT26 cells were cultured at a concentration of 5 × 10^5^ cells per well (6‐well plates) and were incubated for 24 h. Cells were treated with Lig@O‐P or Oxp for 6 h. The cells were then incubated at 37°C for 30 min with the DCFH‐DA probe (Beyotime, China) and detected by FCM (BD, Bioscience). GSH and GSSG assay kit (Beyotime S0053) was used for GSH assay.

### 5.6 Molecular Validation of Nano‐FOLFOX

The CRT and HMGB1 were determined as published. In brief, CT26 cells were grown (1 × 10^5^/well) in coverslips (ThermoFisher) for 24 h. Then the cells were treated by Lig@O‐P. After treatments the cells were washed three times with PBS and fixed using 0.25% paraformaldehyde (PFA) for 10 min. Next, used 0.3% TritonX‐100 to permeabilize for 20 mins and blocked by 5 % BSA in PBS for 30 mins. Subsequently, the anti‐CRT antibody (ab2907, Abcam, 1:500) or HMGB1 antibody (ab18256, Abcam) was incubated overnight at 4°C. Following three times PBST (PBS supplemented with 1 ‰ tween‐20) washes, FITC‐tagged secondary antibody (ab150077, Abcam) was incubated in the dark for 2 h at room temperature. After washed again, the cells were stained by the ProLong Gold Antifade Mountant with DAPI (thereafter referred as DAPI; ThermoFisher) and obtained confocal imaging (LSM‐710, Zeiss).

For CETSA assay, the prepared cell protein samples were divided equally into two 1.5 mL Eppendorf tubes. For the temperature gradient, one tube was treated with Lig@O‐P to a final concentration of 20 µM, while the other tube received an equal volume of DMSO as a control. Both samples were incubated at room temperature for 2 h to allow the drug to bind to the target protein. Each group was further divided into six subgroups and heated at 37°C, 48°C, 52°C, 60°C, 64°C, and 68°C for 5 min. For the concentration gradient, samples were treated with the drug at final concentrations of 0, 15, 30, and 60 µM and incubated at room temperature for 2 h. The samples were then diluted with RIPA lysis buffer to the same concentration. Using a PCR machine, the samples were heated at 56°C for 5 min, with a DMSO‐treated group serving as a control. After heating, all samples were subjected to high‐speed centrifugation at 20,000 × g for 10 min at 4°C to separate insoluble components. The supernatant was boiled at 100°C for 5 min and then assessed using Western blot analysis.

SPR assays were conducted on a Biacore T200 instrument (Cytiva) at 25°C. Recombinant human TS was immobilized on a series S CM5 sensor chip via amine coupling chemistry, achieving an immobilization level of approximately 3500 response units (RU). The analyte, HIS‐EHL‐ME, was serially diluted in running buffer (PBST) and injected over the chip surface at a flow rate of 30 µL/min, with a contact time of 60 s and a dissociation time of 60 s. Binding sensograms were recorded in real‐time, and the data were processed using Biacore Insight Evaluation Software (Version 3.0.12). Kinetic parameters were derived by fitting to a 1:1 binding model, and steady‐state affinity was also assessed.

For Western blot analysis, proteins were extracted from cells using RIPA lysis buffer containing protease and phosphatase inhibitors. Protein quantification was conducted using the BCA protein quantification kit (Thermo Fisher Scientific, USA). Equal amounts of protein were separated on 8%–13.5% tris‐GLY gels and transferred to nitrocellulose (NC) membranes. The membranes were blocked with 5% skim milk for 1 h at room temperature and then incubated with the primary antibody overnight at 4°C. After incubation, the protein bands were washed with TBST (TBS supplemented with 0.1% Tween‐20) and then incubated with the appropriate HRP‐conjugated secondary antibody for 1 h at room temperature. The protein bands were washed four times with TBST for 10 min each and imaged using the imaging system (Clinx Science Instruments, China).

### 5.7 Computational Section

All lignin calculations were performed using guaiacylglycerol‐β‐guaiacyl ether (GGE), a widely accepted lignin model compound. The functionalization and all subsequent computational analyses were conducted on this model compound, rather than on a full polymeric lignin structure. Two additional models, ARG‐GGE and ARG‐GGE‐Me, were constructed to represent ARG‐modified and methylated lignin, respectively. To calculate the ESP of lignin with and without modifications, thousands of model clusters were generated using molecular dynamics (MD) simulations with the extended tight‐binding (xTB) program. The clusters were subsequently optimized using the Molclus [38] program to identify the lowest‐energy conformations. Details of the conformational search procedure have been previously described in our work [37]. The optimized conformations were further refined at the B3LYP‐D3/6‐311G* level using the Gaussian 16 [39] software package, within an implicit water solvent environment modeled using the SMD method. Frequency analysis at the same level of theory was conducted to confirm that the conformations exhibited no imaginary frequencies. Single‐point energy calculations were performed at the PWPB95‐D3/def2‐QZVPP [40, 41] level under the SMD model using ORCA 4.2 package [42]. The same procedure was used to optimize the dUMP and lignin complexes.

The ESP for the most stable conformations was computed using Multiwfn 3.8(dev) [43] and visualized using VMD 1.9.4a50 [44]. To analyze non‐covalent interactions between dUMP and lignin, the independent gradient model based on Hirshfeld partitioning (IGMH) [45] was employed. The complexes of TS with lignin models (with and without modification) were optimized using automated docking via the AutoDock suite [46], which employs a genetic algorithm (GA) to explore ligand conformations and estimate binding affinities. TS structures were prepared by removing water molecules, and adding all missing hydrogens. Lignin model structures were geometry‐optimized, and Gasteiger charges were assigned prior to docking. For each ligand, 100 independent docking runs were performed. The GA parameters consisted of a population size of 150, a maximum number of 2.5 × 10^6^ energy evaluations, and a mutation rate of 0.02. The lowest‐energy conformation was selected as the representative binding mode. Because AutoDock uses an empirical free‐energy scoring function rather than molecular dynamics, no explicit time evolution or atomic trajectories were generated. All analyses and visualization of the docked complexes were conducted using AutoDockTools and PyMOL. The interactions between lignin and TS in the optimal complexes were further analyzed using the PLIP web tool [47].

### 5.8 Anticancer Effect of the Lig@O‐P In Vivo

Female BALB/c mice (6–8 weeks old, 18–20 g) were housed under SPF conditions. CT26 murine colorectal carcinoma cells (ATCC CRL‐2638; 1 × 10^6^ cells in 100 µL PBS) were subcutaneously inoculated into the right flank. When tumors reached 100–150 mm^3^ (volume = length × width^2^/2), mice were randomly assigned (*n* = 5–8/group) to different groups.

1. Monotherapy: groups received q3d × 4 i.p. injections of: PBS(Control‐CTL), Lignin, Oxp, and Lig@O‐P were treated with 5 mg/kg platinum.
2. Combination therapy: groups received q3d × 4: PBS (Control‐CTL), 50 mg/kg 5‐Fu, OxP (5 mg/kg Pt) + 5‐Fu (50 mg/kg, 24 h post‐OxP), Lig@O‐P (5 mg/kg Pt) + 5‐Fu (50 mg/kg, 24 h post‐Lig@O‐P), or FOLFOX‐mimic (OxP/Lig@O‐P [5 mg/kg Pt] followed by 5‐Fu [50 mg/kg] 24 h later).

Mice were weighed, and tumor volumes were measured every two days until Day 8 and Day 14, when euthanasia was performed, and further experiments were conducted.

FCM was utilized to analyze the immune system. Following euthanasia, blood, spleen, and tumor samples were harvested and processed into single‐cell suspensions using a 70‐micron filter. Subsequently, 2 microliters of antibody were added, and the samples were analyzed using a flow cytometer (BD Biosciences). For IHC, the tumor tissue was excised, fixed in 4% paraformaldehyde, paraffin‐embedded, and sectioned. The paraffin‐embedded sections were dewaxed using xylene and dehydrated with graded ethanol. The cells were incubated with antibodies, and the nuclei were stained with hematoxylin.

### 5.9 Patient‐Derived Colorectal Cancer Organoid Culture and Drug Evaluation

Fresh CRC tissues obtained from surgery or biopsy were collected in DMEM/F12 containing antibiotics and processed within 24 h. After washing with precooled PBS, tissues were minced into small fragments (approximately 1–2 mm^3^) and digested in DMEM/F12 containing 2% FBS, 50 U mL^−1^ collagenase IV, and 20 U mL^−1^ DNase at 37°C with shaking for 30–45 min. The suspension was filtered through a 100 µm cell strainer, centrifuged at 300 × g for 5 min at 4°C, and washed with DMEM/F12. The resulting cell pellet was resuspended in precooled Matrigel and seeded into pre‐warmed culture plates, followed by solidification at 37°C. Human CRC organoid complete medium was then added, and organoids were cultured at 37°C in a humidified 5% CO2 incubator with medium replacement every 2–4 days.

For drug evaluation, CRC‐PDOs were subjected to five sequential treatments with PBS, free 5‐FU, oxaliplatin + 5‐FU (classical FOLFOX), or Lig@O‐P + 5‐FU, and bright‐field images were recorded during treatment. For ATP‐based viability analysis, organoid‐Matrigel mixtures were seeded in 96‐well plates and treated for 3 days. The culture medium was removed, ATP detection reagent was added directly to each well, and luminescence was measured using a microplate reader. Relative organoid viability was calculated according to the luminescence intensity.

### 5.10 Toxicity Studies of Nano‐FOLFOX in Orthotopic CRC Model

The hemolytic activity of Lig@Oxp‐PD‐L1 was determined using human red blood cell (RBC) concentrate. First, 100 µL of 1*10^6^ RBCs L‐1 concentrate was added to the diluted solutions and incubated at 37°C for 30 min. Cells treated with either PBS or Water for negative and positive control, respectively. In addition, the Lig@Oxp‐PDL1 in different concentrates were added in the RBC solution. Then, the suspensions were centrifuged at 1500 rpm for 10 min. Finally, the photographs were taken to determine the dissolution of blood in the supernatant. In order to further assess toxicity, mice with tumors were treated as described (*n* = 3). Body weight was recorded regularly, and the whole blood and the serum of animals were obtained on Day 35. Moreover, major organs were analyzed using hematoxylin and eosin (H&E) stain experiments.

### 5.11 Statistical Analysis

All experiments were conducted in triplicate, and the data were analyzed using GraphPad Prism software (version 10.0). The results are presented as mean± standard deviation (SD) in this study. The results of all experiments were statistically analyzed using the two‐tailed Student's *t*‐test. Significance among three or more groups was assessed using two‐way ANOVA with Bonferroni's post‐hoc test. Statistical significance was denoted as *p < 0.05, **p < 0.01, ***p < 0.001, and ****p < 0.0001.

## Author Contributions

**L.C**., **W.H**., and **Y.Q**. designed the study. **S.S**. and **L.C**. analyzed the data. **W.H**., **Y.Q**., and **Y.T**. supervised the study. **S.S**., **X.L**., and **T.L**. performed data preprocessing. **S.S**. and **T.L**. implemented statistical analysis. **X.L**. and **C.W**. developed the mice model. **J.Y**., **D.X**., **J.M**., and **Y.M**.conducted the data analysis. **L.H**. and **X.L**. assisted editing and sorted data. J.Z. and J.M. completed experiments during the revision process. **L.C**. and **S.S**. drafted the manuscript. **X.L**., **Y.Q**., and **W.H**. revised the manuscript.

## Funding

The authors disclosed receipt of the following financial support for the research, authorship, and/or publication of this article: National Key Research And Development Plan (2022YFC3401000), National Natural Science Foundation of China (82472087,92359302,22378076), Guangdong Basic and Applied Basic Research Foundation (2024A1515030129,2021B1515230009,2021A1515111169), Guangdong Provincial Key Areas R&D Programs of “Precision medicine and stem cells” (2023B1111020005), the Natural Science Foundation for Outstanding Youth Team Project of Guangdong Province (2024B1515040030).

## Ethics Approval and Consent to Participate

The use of human tumor specimens in this study was approved by the Institutional Ethics Committee at the First Affiliated Hospital of Sun Yat‐sen University (Ethical Code: [2024]282). All procedures involving human specimens were conducted in accordance with the ethical standards of the responsible institutional committee and with the 1964 Helsinki Declaration and its later amendments or comparable ethical standards. All animal experiments reported in this study were approved by Clinical Research and Animal Trials of the First Affiliated Hospital of Sun Yat‐sen University (reference number [2023]343) and were performed in accordance with institutional guidelines for the care and use of laboratory animals.

## Conflicts of Interest

The authors declare no conflicts of interest.

## Supporting information




**Supporting File**: adma74567‐sup‐0001‐SuppMat.docx.

## Data Availability

The data that support the findings of this study are available from the corresponding author upon reasonable request.
