# Supplementary material for: Lignin Mimicking Protein Methylation Augments the Efficacy of FOLFOX Chemotherapy by Anchoring Thymidylate Synthase
Source: Adv Mater. 2026 Aug 17;38(57):e07040. doi: 10.1002/adma.202507040 (PMC13660821; doi:10.1002/adma.202507040)
Supplement: Supplementary file 1 [file ADMA-38-e07040-s001.docx]

**Supporting Information**

**Lignin mimicking protein methylation augments the efficacy of FOLFOX chemotherapy by anchoring thymidylate synthase**

*Shiyao Song, Yan-Lai Tang, Xiaoting Liu, Jiayi Zheng, Chong Wu, Tang Li, Junhong Ye, Dianmin Xia, Yong Mei, Jingxuan Ma, Libin Huang, Xuequn Luo, Liheng Chen***, Yanlin Qin***, Weiling He**

**Supplementary Figures**

**Fig** **S1**

**Supplementary Fig 1.** the electrostatic potential (ESP) distribution of GGE(a), ARG-GGE(b), ARG-GGE-Me(c).

**Fig S2**

**Supplementary Fig 2.** The FTIR spectra of amino acids and methylation modified Lignin.

**Fig S3**

**Supplementary Fig 3.** GPC/SEC characterization of the molecular weight distribution of lignin before and after modification.

**Fig S4**

**Supplementary Fig 4.** HSQC NMR spectra of modified lignin.

**Fig S5**

**Supplementary Fig 5.** (a-c) The average diameter and polydispersity index of methylation modified lignin in different pH value;

**Fig S6**

**Supplementary Fig 6. hydrated particle size in different reaction conditions.** (a) The different reaction organic solvent, (b) the different TMP value, (c) the different NaOH quality, and (d) the different arginine quality in the reaction.

**Fig S7**

**Supplementary Fig 7.** (a) FT-IR spectra of Oxp, Arg-EHL-Me, and 0.5Arg-EHL-Me@Oxp; (b) The Pt concentrate of Lignin (CTL) and LOP by ICP-MS analysis.

**Fig S8**

**Supplementary Fig 8：**SEM images of Arg-EHL-Me (a), Lig @ Oxp (b) and Lig@Oxp-PDL1 (Lig@O-P) (c).

**Fig S9**

|  | **Encapsulation Efficiency** |
| --- | --- |
| **Arg-EHL-Me @Oxp – PDL1 (Lig@O-P)** | **44%** |

**Supplementary Fig 9.** (a) Standard curves of the oxp by HPLC; (b) Encapsulation Efficiency of the oxp in the Lig@O-P.

**Fig S10**

**Supplementary Fig 10.** Hydrodynamic diameter and PDI of Lig@O-P in 10% FBS-containing culture medium.

**Fig S11**

**Supplementary Fig 11.** Fluorescence-based evaluation of pH-dependent anti-PD-L1 retention on Lig@O-P. Fluorescence-labeled anti-PD-L1-coated Lig@O-P was incubated at pH 7.4 or pH 5.5 for 24 h, followed by centrifugation (30000 rpm, 30 min) to collect the nanoparticle-associated fraction. The fluorescence signal associated with the collected fraction was lower after incubation at pH 5.5 than at pH 7.4, supporting reduced surface-retained antibody and acid-promoted surface de-shielding under acidic conditions.

**Fig S****12**

**Supplementary Fig 12.** Representive images for Intracellular fluorescence in Lignin and Lig@O-P treatment groups in different time. Red: Lig@O-P or Lignin (Rhodamine-B), Blue: DAPI, nucleus. Scale bar: 200μm

**Fig S13**

**Supplementary Fig 13.** FCM analysis of intercellular uptake of Lig@O-P in HCT116 cells.

**Fig S14**

**Supplementary Fig 14.** Pt concentration in cell for CTL, Oxp, Lig@Oxp and Lig@O-P.

**Fig S15**

**Supplementary Fig 15.** Statistical Results of the (a) Live/Dead; (b) Apoptosis; (c) commet assay; and (d) cell cycle.

**Fig S16**

**Supplementary Fig 16.** (a) FCM results of ROS assay by DCFH probe on DMSO, 30μM Oxp and Lig@O-P; (b) Statistical Results of the ROS in Fluorescence microscopy image; (c) GSH results of the different treatment.

**Fig S17**

**Supplementary Fig 17.** Western blotting of PARP, and CyclinD1 in CT26 cells after the indicated treatment by 30μM Oxp, Lignin and Lig@O-P for 24 h.

**Fig S18**

**Supplementary Fig 18.** Interaction between TS and lignin with different treatment.

**Fig S19**

**Supplementary Fig 19.** Interaction between dUMP and lignin with different treatment.

**Fig S20**

**Supplementary Fig 20.** The (a) Bliss_Loewe, (b) HAS, (c) Bliss, (d) loewe score in SynergyFinder 2.0.

**Fig S21**

**Supplementary Fig 21.** Representive Confocal Fluorescence microscopy image of the CRT and HMGB1 IF assay.

**Fig S22**

**Supplementary Fig 22.** Enzyme activity inhibition experiment: The concentration of dTMP production varies with the addition concentration of Lig@O-P.

**Fig S23**

**Supplementary Fig 23**. Evaluation of the Lig@O-P-based FOLFOX strategy in patient-derived colorectal cancer organoids (CRC-PDOs). Representative bright-field images of CRC-PDOs subjected to four sequential treatments with PBS, free 5-FU, oxaliplatin + 5-FU (classical FOLFOX), or Lig@O-P + 5-FU, with images acquired every 12 h over 3 days.
